# Synthesis and spectroscopic properties of β-*meso* directly linked porphyrin–corrole hybrid compounds

**DOI:** 10.3762/bjoc.14.13

**Published:** 2018-01-22

**Authors:** Baris Temelli, Hilal Kalkan

**Affiliations:** 1 Hacettepe University, Department of Chemistry, Beytepe Campus, 06800, Ankara, Turkey

**Keywords:** corrole, hybrid compounds, indium(III) chloride, porphyrin, porphyrinoids

## Abstract

The preparation of β-*meso* directly linked porphyrin–corrole hybrids was realized for the first time via an InCl_3_-catalyzed condensation reaction of 2-formyl-5,10,15,20-tetraphenylporphyrins with *meso*-substituted dipyrromethanes. Hybrid compounds have been characterized by ^1^H NMR, ^13^C NMR, 2D NMR, UV–vis absorption and fluorescence spectroscopy.

## Introduction

**Scheme 1:**
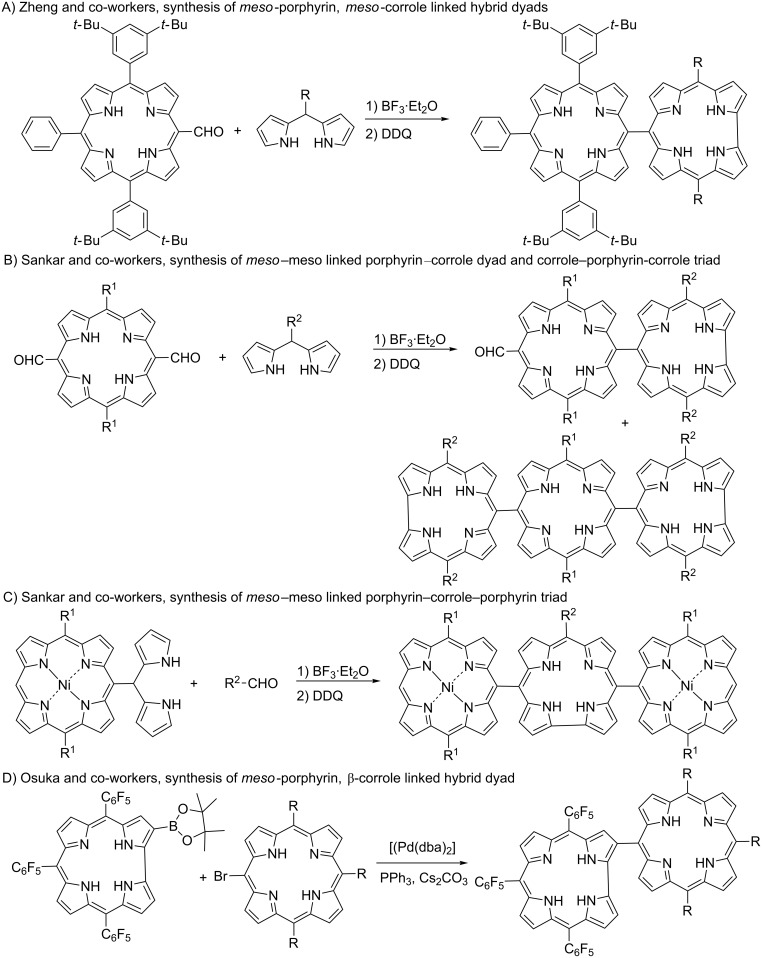
Overview of the synthesis of directly linked porphyrin–corrole hybrid compounds.

Porphyrins and metalloporphyrins play an important role in chemistry, biology, medical and materials sciences because of their presence in biological compounds such as chlorophyll and heme molecules that have very important functions in the metabolism of living organisms [1–2]. In recent years, efforts in porphyrin chemistry have been focused on the synthesis of multichromophore containing compounds and their potential applications in molecular wires, sensors, nonlinear optical devices, photosensitizers and organic conducting materials [3–6]. The first studies on the synthesis of multichromophores were based on obtaining multiporphyrin arrays [7–9] that could stabilize only metal ions in a bivalent state. To overcome this limitation, porphyrin conjugates with different chromophore groups such as fullerene [10–12], BODIPY [13–15], corrole [16–23], phthalocyanine [24–26], subporphyrin [27] and expanded porphyrins [28–30] were prepared and their photophysical and electrochemical properties were characterized. Among these compounds, corroles, contracted porphyrin analogues [31–33], assumed an important place in porphyrin chemistry due to their small cavities, trianionic characters, high fluorescence quantum yields and favorable electronic properties. Porphyrin–corrole conjugates have been successfully used as donor–acceptor systems in photoinduced charge separation processes. It was shown that derivatives of these conjugates could be potentially used in photovoltaic applications [21–23]. In order to achieve rapid energy and electron transfer between macrocycles, the short distance between subunits keeps an important place. Therefore, two important factors affect the physical and electronic properties of porphyrin–corrole conjugates: (i) type of linkers and (ii) position of substitution (*meso* or β). So far, corrole macrocyles have been integrated into porphyrin conjugates via anthracene, biphenylene, xanthene, dibenzofuran [16–20], amide [21] and triazole [22–23] linkers. Despite the large number of studies on the synthesis of porphyrin–corrole conjugates, systematic studies on directly linked porphyrin–corrole hybrid structures are limited and there is no report on β-porphyrin, *meso*-corrole linked hybrids of these conjugates. Zheng et al. synthesized *meso*–*meso* directly linked porphyrin–corrole dyads by the condensation reaction of *meso*-formylated porphyrin with *meso*-substituted dipyrromethanes (Scheme 1A) [34]. Later, Sankar et al. used the same type of condensation reaction to obtain *meso*–*meso* linked corrole–porphyrin–corrole (Scheme 1B) [35] and porphyrin–corrrole–porphyrin triads (Scheme 1C) [36]. The only report on the synthesis of *meso*-porphyrin, β-corrole-linked hybrid structure described a Suzuki–Miyaura cross-coupling reaction between a β-borylated corrole and *meso*-bromoporphyrins (Scheme 1D) [37].

**Scheme 2:**
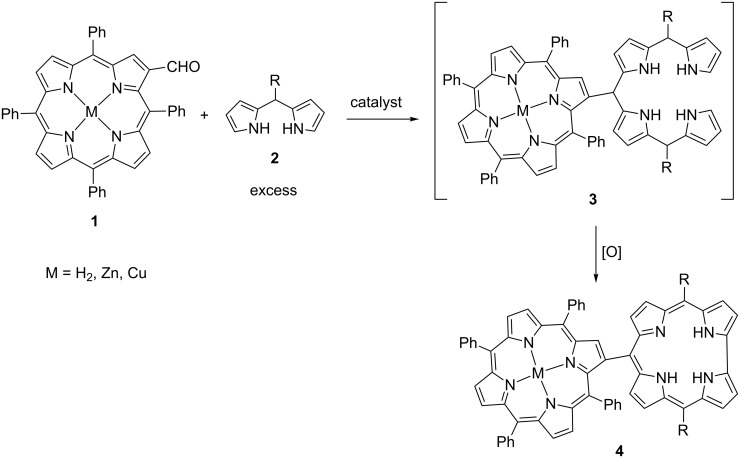
Synthesis of β-*meso* directly linked porphyrin–corrole hybrid compounds.

Recently, we have successfully synthesized *meso*–*meso* and β-*meso*-linked imine-bridged porphyrin–corrole conjugates and investigated intramolecular energy transfer between macrocycles [38]. As a part of our ongoing research on porphyrin–corrole conjugates, herein we describe a convenient synthesis of a series of novel directly linked porphyrin–corrole hybrid compounds. For this purpose, acid-catalyzed reactions of dipyrromethanes and aldehydes, which have been used frequently for the synthesis of *meso*-substituted corroles, were carried out [31,39–40]. The key bilane-substituted porphyrin intermediates **3** were obtained by the addition of an excess amount of *meso*-substituted dipyrromethanes **2** to β-formylated porphyrins **1** and these intermediates were oxidized in the reaction medium to form the desired hybrid structures **4** (Scheme 2).

## Results and Discussion

Initially, the reaction of 2-formyl-5,10,15,20-tetraphenylporphyrin (**1**) with 4 equivalents of phenyldipyrromethane (**2a**) was tested using *p*-toluenesulfonic acid (TsOH) as catalyst at room temperature but no product was observed in the reaction (Table 1, entry 1). Increasing the reaction temperature affected the production of **4a** and the target compound could be obtained with 6% yield (Table 1, entries 2–4). A screen of solvents revealed that chloroform provided the best yield and was superior to 1,2-dichloroethane, toluene and methanol (Table 1, entries 3–6). The ratio of **1a**/**2a** played a little role in improving the yield of product (Table 1, entries 7–9). The yield of **4a** increased slightly to 7% when the reaction was carried out with 6 equiv of **2a** (Table 1, entry 8). Further increasing the amount of **2a** led to the formation of unidentified byproducts in the reaction medium and a reduction of the yield of **4a** (Table 1, entry 9). Then, the activities of different catalysts, varying from clay catalysts to Lewis acids, were tested in the model reaction (Table 1, entries 10–19). AgOTf, AlCl_3_, ZnBr_2_ and InCl_3_-catalyzed the reaction in higher yields with an unexpected byproduct, *meso*–β-substituted directly linked porphyrin–porphyrin dyad **5a** (Table 1, entries 16–19). The structure of **5a** was confirmed by comparison with literature data [41]. The highest yield of **4a** was obtained with InCl_3_ (19%, Table 1, entry 19). Other catalysts gave no (Table 1, entries 10 and 11) or lower yields of products (Table 1, entries 12–15). When the reaction temperature increased to 80 °C by using 1,2-dichloroethane as solvent in the presence of InCl_3_ as catalyst, the product yield decreased to 14% (Table 1, entry 20).

**Table 1:** Optimization of the reaction conditions.

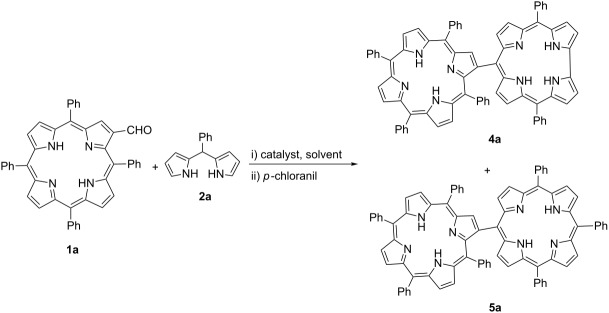						

Entry	**1a**/**2a**	Solvent	Catalyst	Temp (°C)	**4a** (%)^a^	**5a** (%)^a^

1	1:4	CHCl_3_	TsOH^b^	rt	–	–
2	1:4	CHCl_3_	TsOH^b^	40	–	–
3	1:4	CHCl_3_	TsOH^b^	60	6	–
4	1:4	ClCH_2_CH_2_Cl	TsOH^b^	60	5	–
5	1:4	MeOH	TsOH^b^	60	2	–
6	1:4	toluene	TsOH^b^	60	4	–
7	1:2	CHCl_3_	TsOH^b^	60	1	–
8	1:6	CHCl_3_	TsOH^b^	60	7	–
9	1:10	CHCl_3_	TsOH^b^	60	4	–
10	1:6	CHCl_3_	Mont. K-10^c^	60	–	–
11	1:6	CHCl_3_	BF_3_·OEt_2_^d^	60	–	–
12	1:6	CHCl_3_	Mont. KSF^c^	60	2	–
13	1:6	CHCl_3_	Cu(OTf)_2_^b^	60	4	–
14	1:6	CHCl_3_	Amberlyst-15^c^	60	3	–
15	1:6	CHCl_3_	TFA^b^	60	8	–
16	1:6	CHCl_3_	AgOTf^b^	60	17	3
17	1:6	CHCl_3_	AlCl_3_^d^	60	14	3
18	1:6	CHCl_3_	ZnBr_2_^b^	60	15	2
19	1:6	CHCl_3_	InCl_3_^b^	60	19	4
20	1:6	ClCH_2_CH_2_Cl	InCl_3_^b^	80	14	3

^a^Isolated yield after column chromatography. Catalyst amount: ^b^10 mol %, ^c^1 mmol aldehyde/1 g of catalyst, ^d^100 mol %.

**Scheme 3:**
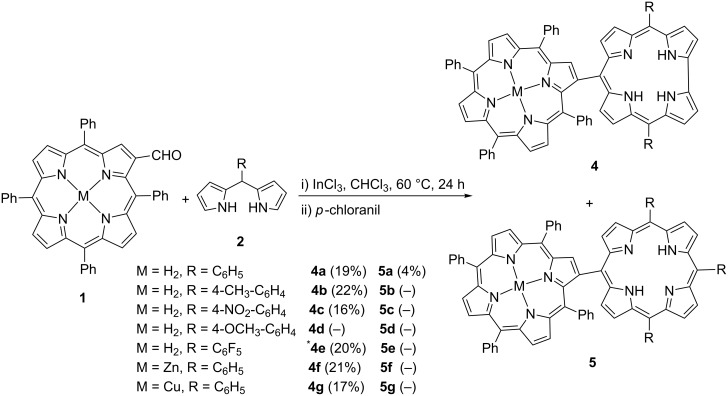
Synthesis of porphyrin–corrole hybrid derivatives*.* *100 mol % of AlCl_3_ was used as a catalyst.

With the optimized reaction conditions in hand, different *meso*-substituted dipyrromethanes **2** were subjected to the condensation reactions (Scheme 3). Electronic properties of the substituents on the dipyrromethane generally did not show significant effects on the reactions except for the electron-donating methoxy substituent (Scheme 3, **4d**), which resulted in a mixture of unidentified compounds after the reaction. This might be due to an acid-catalyzed rearrangement of the substituents, called scrambling, which is a common problem in porphyrin synthesis in the reaction of dipyrromethanes with aldehydes [42]. When electron-withdrawing 4-nitrophenyl and pentafluorophenyl substituents were used on the dipyrromethane, hybrid compounds **4c** and **4e** were isolated in 16% and 20% yields, respectively. The synthesis of **4e** with InCl_3_ afforded a very low yield, thus an equimolar amount of AlCl_3_ was used to obtain this product. In order to expand the scope of the reaction, metal complexes of formylated porphyrins were subjected to the reaction. While Zn-porphyrin containing hybrid compound **4f** was isolated in 21%, the Cu(II) complex of β-formylated porphyrin underwent the reaction to produce the hybrid compound **4g** in 17% yield (Scheme 3).

**Figure 1:**
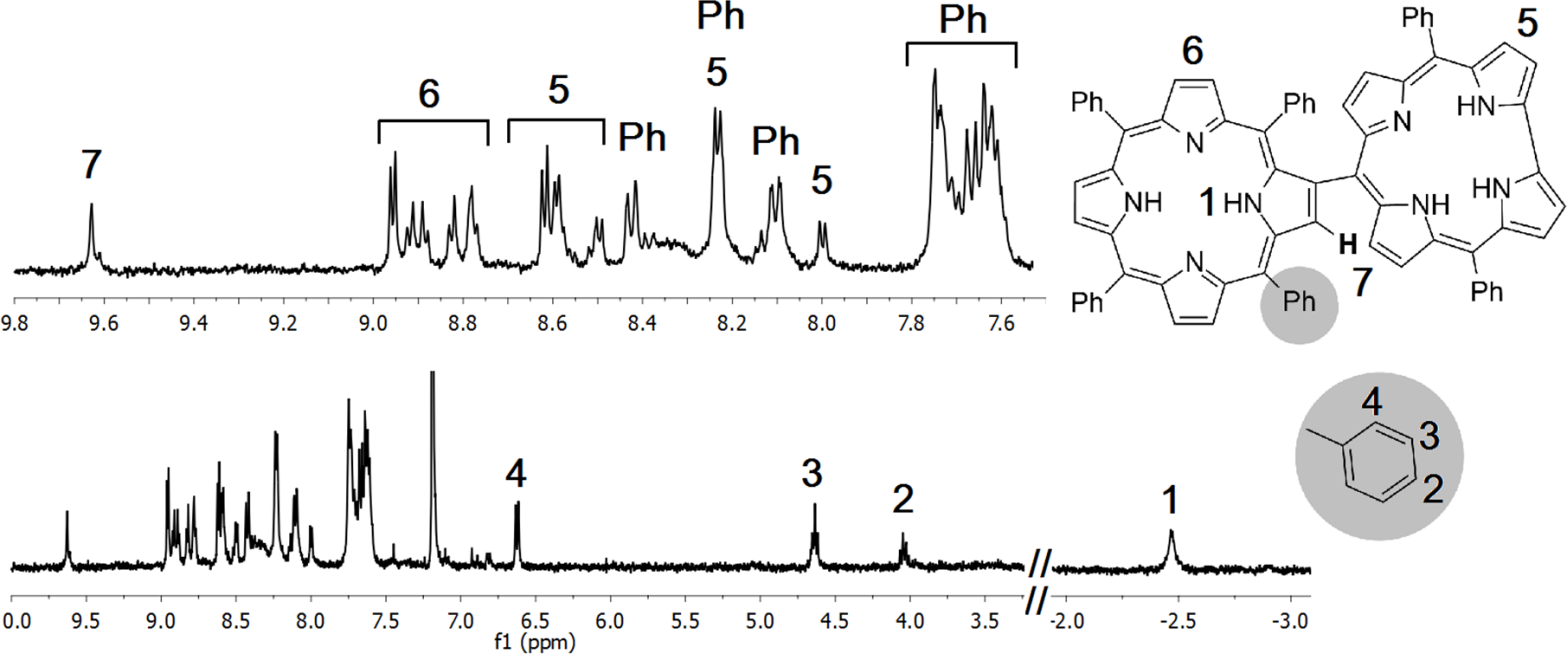
^1^H NMR spectrum of **4a** in CDCl_3_.

The structures of the hybrid compounds were identified by using ^1^H NMR, ^13^C NMR, ^1^H,^1^H-COSY NMR and HRMS techniques (see Supporting Information File 1). The ^1^H NMR spectrum of **4a** is shown in Figure 1. As reported for similar dimeric porphyrin systems, one of the *meso*-linked phenyl substituents on the porphyrin core was located above the corrole macrocycle and was affected by the ring current. These phenyl protons appear strongly shifted toward higher field. The positions of the protons were assigned by comparing previously reported β–*meso*-substituted porphyrin arrays [41]. The *para*-proton (2), the *meta*-protons (3) and the *ortho*-protons (4) of the phenyl substituent appeared at 4.05 ppm, 4.64 ppm and 6.62 ppm, respectively. The β-protons of the porphyrin and the corrole macrocycles gave multiplets and doublets between 8.00 and 10.00 ppm as expected. Inner N–H protons of the corrole macrocycle gave a very broad signal and could not be determined due to its high unsymmetrical property and an excess amount of tautomeric structures. The N–H protons of the porphyrin macrocycle resonated at −2.46 ppm (Supporting Information File 1, Figure S1).

The absorption spectra of hybrid compounds have been recorded and λ_max_ values are listed with molar extinction coeffients (ε) in Table 2. Split Soret bands were observed for **4b**, **4e** and metalloporphyrin **4g**. These results indicate a strong exciton coupling between the neighbor macrocycles in hybrid structures. The same effect was also observed in the emission spectra. Fluorescence spectra of the compounds gave split emission bands except for **4c**. Fluorescence life times and quantum yields were further determined for the hybrid compounds (Table 2). Measured fluorescence life times (4–9 ns), and fluorescence quantum yields are in agreement with the literature data [35–36]. Strong electron-withdrawing pentafluorophenyl-substituted **4e** and Zn–porphyrin complex **4f** gave higher quantum yields compared to reported literature values for *meso*–*meso*-linked porphyrin–corrole dyads [34] and traids [35]. High fluorescence quantum yield properties of the new hybrid compounds are promising for many applications such as organic optoelectronics or biological imaging.

**Table 2:** Photophysical properties of hybrid compounds.

Compound	λ_abs_/nm (ε/10^−5^ M^−1^·cm^−1^)^a^	λ_em_/nm^b^	Φ^c^	τ/ns^d^

**4a**	421 (5.35), 520 (4.34), 552 (4.08), 595 (4.03), 652 (3.92)	664, 723	0.10	7.4
**4b**	421 (5.32), 441 (4.96), 523 (4.18), 559 (4.08), 597 (4.04), 659 (3.53)	664, 725	0.22	7.1
**4c**	421 (4.94), 518 (4.08), 596 (3.90), 662 (3.81)	692	0.04	8.5
**4e**	420 (5.21), 443 (5.07), 521 (4.32), 554 (4.10), 591 (4.06), 646 (3.86)	663, 717	0.27	5.4
**4f**	421 (4.57), 442 (4.46), 524 (3.49), 553 (3.64), 657 (2.98)	663, 728	0.41	7.8
**4g**	418 (4.65), 438 (4.47), 542 (3.68), 656 (2.94)	651, 720	0,01	4.4

^a^Absorption spectra were recorded in CHCl_3_. ^b^Fluorescence spectra were recorded in CHCl_3_ (λ_ex_: 420 nm). ^c^Based on TPP in toluene (Φ = 0.11). ^d^Excited at 390 nm.

## Conclusion

In summary, we have synthesized the first examples of β-porphyrin, *meso*-corrole substituted directly linked porphyrin–corrole hybrid compounds via a condensation reaction of *meso*-substituted dipyrromethanes with β-formylated *meso*-tetraphenylporphyrins. Spectroscopic and photophysical properties of the new hybrid structures have been determined. Higher quantum yields of some of the synthesized hybrid compounds indicate that these compounds can be considered as good candidates for many important applications ranging from photovoltaics to medicine.

## Supporting Information

File 1 General experimental information, experimental details on the synthesis of products **4** and **5a**, and full characterization data of all products.
