# Supplementary material for: Synthesis and spectroscopic properties of β-meso directly linked porphyrin–corrole hybrid compounds
Source: Beilstein J Org Chem. 2018 Jan 22;14:187–93. doi: 10.3762/bjoc.14.13 (PMC5789387; doi:10.3762/bjoc.14.13)
Supplement: File 1 — General experimental information, experimental details on the synthesis of products 4 and 5a, and full characterization data of all products. [file Beilstein_J_Org_Chem-14-187-s001.pdf]

Supporting Information

for

Synthesis and spectroscopic properties of β–*meso* directly linked porphyrin–corrole hybrid compounds

Baris Temelli* and Hilal Kalkan

Address: Hacettepe University, Department of Chemistry, Beytepe Campus, 06800, Ankara, Turkey

Email: Baris Temelli - temelli@hacettepe.edu.tr

* Corresponding author

General experimental information, experimental details on the synthesis of products 4 and 5a, and full characterization data of all products

Experimental section

General methods. All reactions were performed under N_2 atmosphere. All reagents and solvents were of reagent grade. The NMR spectra were recorded in $CDCl_3$ on a 400 MHz instrument with TMS as the internal standard. NMR data are represented as follows: chemical shift (ppm), multiplicity (s = singlet, brs = broad singlet, d = doublet, t = triplet, q = quartet, m = multiplet), coupling constants in hertz (Hz). IR spectra were recorded on FTIR spectrometer. HRMS were measured in ESI mode and the mass analyzer of the HRMS was TOF. Flash column chromatography was performed on silica gel (230–400 mesh). β-Formylated 5,10,15,20-

tetraphenylporphyrins **1** [1] and *meso*-substituted dipyrromethanes [2] were prepared according to literature methods and their spectral data matched literature values.

General procedure for preparing hybrid compounds. To a solution of compound **1** (0.100 mmol) and **2** (0.600 mmol, 6.0 equiv) in $CHCl_3$ (5 mL) under N_2 atmosphere was added $InCl_3$ (0.010 mmol, 10mol %) (0.100 mmol, 100 mol % $AlCl_3$ for **4d**) and the mixture was stirred at 60 °C for 24 h. The reaction mixture cooled to room temperature and *p*-chloranil (0.150 mmol, 1.5 equiv) was added. After 12 h. the reaction mixture was quenched with H_2O (5 mL) and aqueous phase was extracted with $CHCl_3$. Solvent was removed under reduced pressure and crude product was purified by flash column chromatography over silica gel with EtOAc:*n*-hexane (1:10).

Compound **4a**. 20 mg, 19% yield, purple solid. mp > 300 °C; R_f : 0.44 (1:10 EtOAc/n-hexane); FTIR (ATR): 669, 701, 726, 752, 801, 880, 965, 979, 1002, 1074, 1126, 1176, 1263, 1374, 1464, 1542, 1559, 1598, 1634, 1654, 1683, 1699, 1716, 2851, 2924, 2955 cm^{-1}; ^1H NMR (400 MHz, $CDCl_3$): δ -2,46 (brs, 2H, N-H), 4.05 (t, *J*= 7.3 Hz, 1H, *p*-20^4, Ar-H), 4.64 (t, *J*= 7.4 Hz, 2H, *m*-$20^{3,5}$, Ar-H), 6.62 (d, *J*= 7.2 Hz, 2H, *o*-$20^{2,6}$, Ar-H), 7.60-7.80 (m, 18H, Ar-H), 8.00 (d, *J*= 5.0 Hz, 1H, porphyrin *β*-H), 8.08-8.12 (m, 2H, Ar-H), 8.20-8.27 (m, 5H, 3 Ar-H, 2 corrole *β*-H), 8.42 (d, *J*= 7.0 Hz, 2H, Ar-H), 8.50 (d, *J*= 5.0 Hz, 1H, porphyrin *β*-H), 8.59 (d, *J*= 5.0 Hz, 2H, corrole *β*-H), 8.62 (d, *J*= 4.8 Hz, 2H, corrole *β*-H), 8.75-8.79 (m, 1H, porphyrin *β*-H), 8.80-8.84 (m, 1H, porphyrin *β*-H), 8.86-8.89 (m, 1H, porphyrin *β*-H), 8.90-8.93 (m, 1H, porphyrin *β*-H), 8.96 (d, *J*= 4.3 Hz, 2H, corrole *β*-H), 9.63 (s, 1H, pophyrin *β*-H); ^{13}C NMR (100 MHz, $CDCl_3$) δ 120.6, 126.9, 127.4, 128.0, 128.2, 129.0, 134.5, 134.6, 134.7, 135.0, 141.6, 141.8, 142.0, 142.5; HRMS (ESI) *m/z* calcd for $C_{75}H_{51}N_8$ $(M+H)^+$ 1063.4231, found 1063.4212.

Compound **4b**. 24 mg, 22% yield, purple solid. mp > 300 °C; R_f : 0.46 (1:10 EtOAc/n-hexane); FTIR (ATR): 665, 701, 728, 750, 806, 880, 974, 962, 998, 1071, 1130, 1181, 1265, 1374, 1460, 1537, 1552, 1601, 1639, 1651, 1683, 1700, 2863, 2920, 2968 cm^{-1}; ^1H NMR (400 MHz, $CDCl_3$): δ -2,90 (brs, 2H, N-H), -2.45 (brs, 1H, NH), 2.62 (s, 3H, CH_3), 2.65 (s, 3H, CH_3), 3.90-3.96 (m, 1H, *p*-20^4, Ar-H), 4.60-4.67 (m, 2H, *m*-$20^{3,5}$, Ar-H), 6.80 (d, *J*= 6.9 Hz, 2H, *o*-$20^{2,6}$, Ar-H), 7.42-7.50 (m, 6H), 7.60-7.75 (m, 7H), 7.95-8.03 (m, 4H), 8.10-8.15 (m, 4H), 8.20-8.25 (m, 2H), 8.35-8.40 (m, 2H), 8.51-8.57 (m, 4H), 8.75-8.83 (m, 6H), 8.87-8.93 (m, 2H), 9.61 (s, 1H, pophyrin

β-H); ^{13}C NMR (100 MHz, $CDCl_3$) δ 29.0, 29.4, 118.9, 119.1, 120.3, 120.8, 122.3, 123.5, 124.0, 124.4, 124.5, 126.7, 126.8, 126.9, 127.4, 127.8, 128.9, 131.0, 132.0, 132.4, 137.2, 138.5, 138.9, 139.4, 139.5, 142.0, 142.2, 142.5, 145.5, 147.1, 147.7, 149.2; HRMS (ESI) *m/z* calcd for $C_{77}H_{55}N_8$ $(M+H)^+$ 1091.4544, found 1091.4560.

Compound **4c**. 18 mg, 16% yield, purple solid. mp > 300 °C; R_f : 0.89 ($CHCl_3$); FTIR (ATR): 669, 701, 726, 751, 799, 846, 865, 884, 964, 980, 1002, 1073, 1107, 1178, 1223, 1267, 1336, 1375, 1464, 1516, 1540, 1558, 1592, 1717, 2852, 2925, 2955 cm^{-1}; ^{1}H NMR (400 MHz, $CDCl_3$): δ -2.40 (brs, 2H, N-H), 4.07-4.14 (brs, 1H, *p*-20^4, Ar-H), 4.68 (t, *J*= 7.2 Hz, 2H, *m*-$20^{3,5}$, Ar-H), 6.69 (d, *J*= 7.8 Hz, 2H, *o*-$20^{2,6}$, Ar-H), 7.50-7.56 (m, 1H, Ar-H), 7.67-7.86 (m, 12H, 10 Ar-H, 2 corrole *β*-H), 8.04-8.09 (m, 1H, porphyrin *β*-H), 8.14-8.20 (m, 2H, Ar-H), 8.27-8.33 (m, 2H, corrole *β*-H), 8.40-8.45 (m, 2H, Ar-H), 8.48-8.54 (m, 2H, Ar-H), 8.56-8.73 (m, 9H, 6 Ar-H, 2 corrole *β*-H, 1 porphyrin *β*-H), 8.82-8.86 (m, 1H, porphyrin *β*-H), 8.87-8.91 (m, 1H, porphyrin *β*-H), 8.98-9.03 (m, 2H, porphyrin *β*-H), 9.09-9.16 (m, 2H, corrole *β*-H), 9.71 (s, 1H, porphyrin *β*-H); ^{13}C NMR (100 MHz, $CDCl_3$) δ 120.5, 120.8, 121.9, 123.4, 126.7, 126.8, 127.9, 128.9, 130.9, 131.0, 132.3, 133.9, 134.5, 134.7, 134.9, 135.4, 141.8, 142.1, 144.0, 147.0; HRMS (ESI) *m/z* calcd for $C_{75}H_{48}$ $N_{10}O_4$ $(M-H)^-$ 1151.3787, found 1151.3717.

Compound **4e**. 25 mg, 20% yield, purple solid. mp > 300 °C; R_f : 0.51 (1:1 $CHCl_3$/n-hexane); FTIR (ATR): 668, 705, 753, 802, 929, 981, 1057, 1214, 1263, 1378, 1481, 1527, 1718, 2847, 2923, 2956 cm^{-1}; ^{1}H NMR (400 MHz, $CDCl_3$): δ -2.43 (brs, 2H, N-H), 3.93 (t, *J*= 7.5 Hz, 1H, *p*-20^4, Ar-H), 4.56 (t, *J*= 7.2 Hz, 2H, *m*-$20^{3,5}$, Ar-H), 6.61 (d, *J*= 7.8 Hz, 2H, *o*-$20^{2,6}$, Ar-H), 7.68-7.83 (m, 11H, 9 Ar-H, 2 corrole *β*-H), 8.11 (d, *J*= 4.6 Hz, 1H, porphyrin *β*-H), 8.17 (d, *J*= 7.1 Hz, 2H, Ar-H), 8.29-8.32 (m, 2H, corrole-H), 8.48-8.52 (m, 4H, Ar-H), 8.62 (brs, 2H, corrole *β*-H), 8.67 (d, *J*= 4.7 Hz, 1H, porphyrin *β*-H), 8.96 (d, *J*= 4.7 Hz, 1H, porphyrin *β*-H), 9.00 (d, *J*= 4.8 Hz, 1H, porphyrin β-H), 9.08 (d, *J*= 4.8 Hz, 1H, porphyrin *β*-H), 9.11 (d, *J*= 4.6 Hz, 1H, porphyrin *β*-H), 9.17 (d, *J*= 4.3 Hz, 2H, corrole *β*-H), 9.86 (s, 1H, porphyrin *β*-H); ^{13}C NMR (100 MHz, $CDCl_3$) δ 120.1, 120.4, 120.7, 123.7, 125.2, 125.5, 126.1, 128.9, 129.1, 131.0, 134.0, 134.7, 134.8, 134.9, 135.0, 135.2, 139.1, 139.2, 139.3; HRMS (ESI) *m/z* calcd for $C_{75}H_{41}F_{10}N_8$ $(M+H)^+$ 1243.3289, found 1243.3247.

Compound **4f**. 24 mg, 21% yield, purple solid. mp > 300 °C; R_f : 0.22 (1:10 EtOAc/n-hexane); FTIR (ATR): 701, 759, 802, 969, 999, 1075, 1130, 1278, 1339, 1375, 1457, 1697, 2853, 2926, 2962 cm^{-1}; ^{1}H NMR (400 MHz, $CDCl_3$): δ -2.80 (brs, 2H, N-H), 4.00 (t, *J*= 7.5 Hz, 1H, *p*-20^{4}- Ar-H), 4.71 (t, *J*= 7.6 Hz, 2H, *m*-$20^{3,5}$, Ar-H), 6.88 (d, *J*= 7.6 Hz, 2H, *o*-$20^{2,6}$ Ar-H), 7.65-7.85 (m, 16H), 8.14-8.20 (m, 5H), 8.30 (d, *J*= 4.8 Hz, 1H, porphyrin *β*-H), 8.31-8.34 (m, 4H), 8.43-8.47 (m, 2H), 8.57-8.58 (m, 2H), 8.63 (d, *J*= 4.8 Hz, 2H), 8.71 (d, *J*= 4.6 Hz, 1H, porphyrin *β*-H), 8.86 (brs, 3H), 8.97 (d, *J*= 4.8 Hz, 1H, porphyrin *β*-H), 9.00 (d, *J*= 4.6 Hz, 1H, porphyrin *β*-H), 9.07 (d, *J*= 4.7 Hz, 1H, porphyrin *β*-H), 9.10 (d, *J*= 4.7 Hz, 1H, porphyrin *β*-H), 9.83 (s, 1H, porphyrin *β*-H); ^{13}C NMR (100 MHz, $CDCl_3$) δ 120.9, 121.6, 126.7, 127.7, 127.9, 128.8, 133.0, 133.4, 134.4, 134.5, 134.6, 136.2, 141.6, 142.1, 142.3, 143.2, 146.2, 147.2, 150.1, 150.6, 151.3, 151.7, 151.8; HRMS (ESI) *m/z* calcd for $C_{75}H_{49}N_8Zn$ $(M+H)^+$ 1125.3366, found 1125.3283.

Compound **4g**. 19 mg, 17% yield, purple solid. mp > 300 °C; R_f : 0.30 (1:10 EtOAc/n-hexane); FTIR (ATR): 701, 722, 741, 799, 966, 980, 996, 1004, 1038, 1071, 1122, 1178, 1275, 1345, 1381, 1466, 1724, 2854, 2923, 2955 cm^{-1}; HRMS (ESI) *m/z* calcd for $C_{75}H_{48}CuN_8$ $(M+H)^+$ 1124.3371, found 1124.3335.

Compound **5a**. 4.6 mg, 4% yield, purple solid. mp > 300 °C; R_f : 0.14 (1:1 CH_2Cl_2/n-hexane); FTIR (ATR): 667, 700, 749, 801, 964, 980, 1002, 1032, 1072, 1122, 1174, 1215, 1349, 1441, 1472, 1560, 1597, 1717, 2853, 2926, 3023 cm^{-1}; ^{1}H NMR (400 MHz, $CDCl_3$): δ -2,83 (brs, 2H, N-H), -2,38 (brs, 2H, N-H), 4.04 (t, *J*= 6.8 Hz, 1H, *p*-20^{4}, Ar-H), 4.73 (t, *J*= 7.6 Hz, 2H, *m*-$20^{3,5}$, Ar-H), 6.88 (d, *J*= 6.9 Hz, 2H, *o*-$20^{2,6}$, Ar-H), 7.62-7.83 (m, 18H, Ar-H), 8.14-8.23 (m, 6H, 5 Ar-H, 1 *β*-H), 8.28-8.34 (m, 4H, Ar-H), 8.36-8.41 (m, 1H, Ar-H), 8.44-8.47 (m, 2H, Ar-H), 8.58 (d, *J*= 4.8 Hz, 2H, *β*-H), 8.61-8.66 (m, 2H, *β*-H), 8.83-8.92 (m, 5H, *β*-H), 8.90 (d, *J*= 4.5 Hz, 1H, *β*-H), 8.97 (d, *J*= 4.7 Hz, 1H, *β*-H), 9.00 (d, *J*= 4.7 Hz, 1H, *β*-H), 9.68 (s, 1H, *β*-H); ^{13}C NMR (100 MHz, $CDCl_3$) δ 120.1, 120.3, 120.4, 120.7, 122.5, 122.6, 124.3, 126.8, 127.0, 127.1, 127.6, 127.9, 128.0, 128.2, 132.5, 134.7, 134.8, 135.0, 135.2, 139.3, 142.2, 142.5, 142.6, 142.7; HRMS (ESI) *m/z* calcd for $C_{82}H_{55}N_8$ $(M+H)^+$ 1151.4544, found 1151.4518.

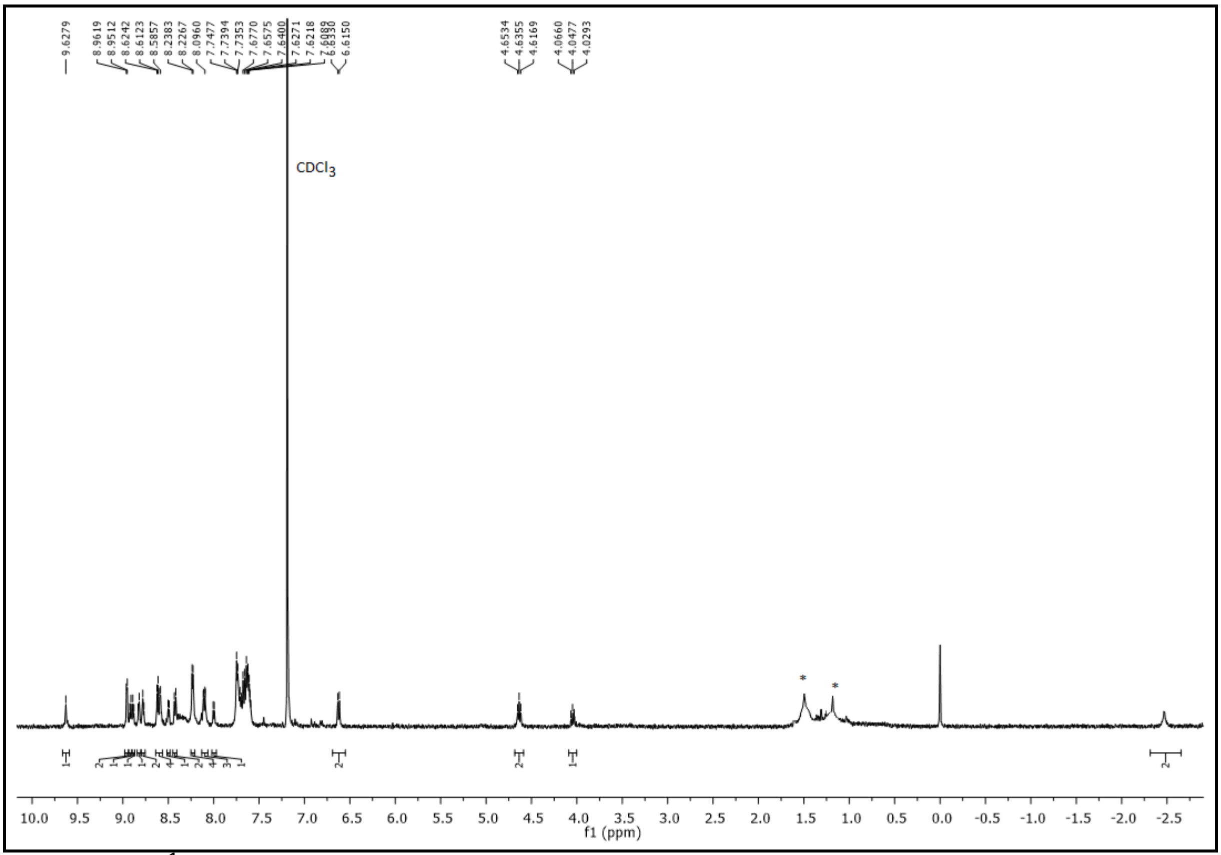

Figure S1. ^{1}H NMR spectrum of **4a** (Solvent: $CDCl_3$) * Solvent impurities

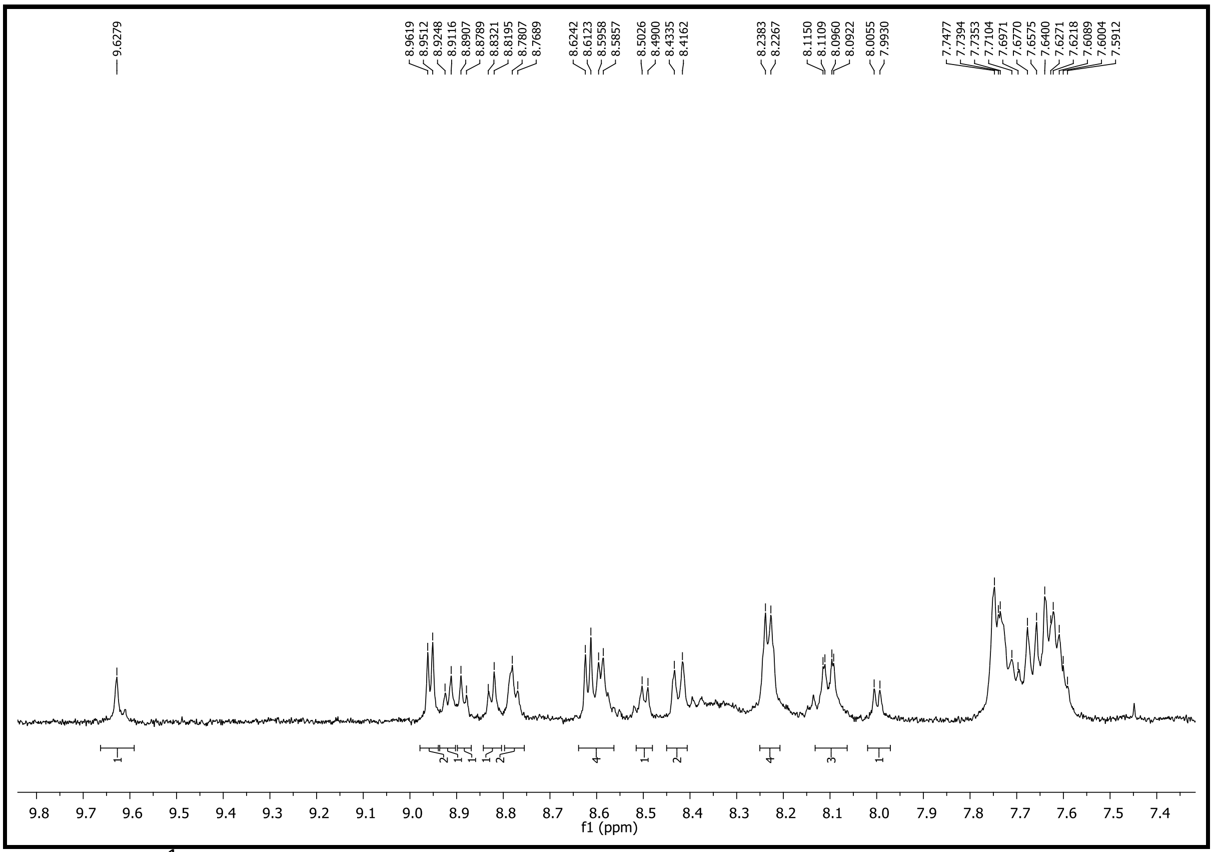

Figure S2. ^{1}H NMR spectrum of **4a** (7.3–9.8 ppm, Solvent: $CDCl_3$)

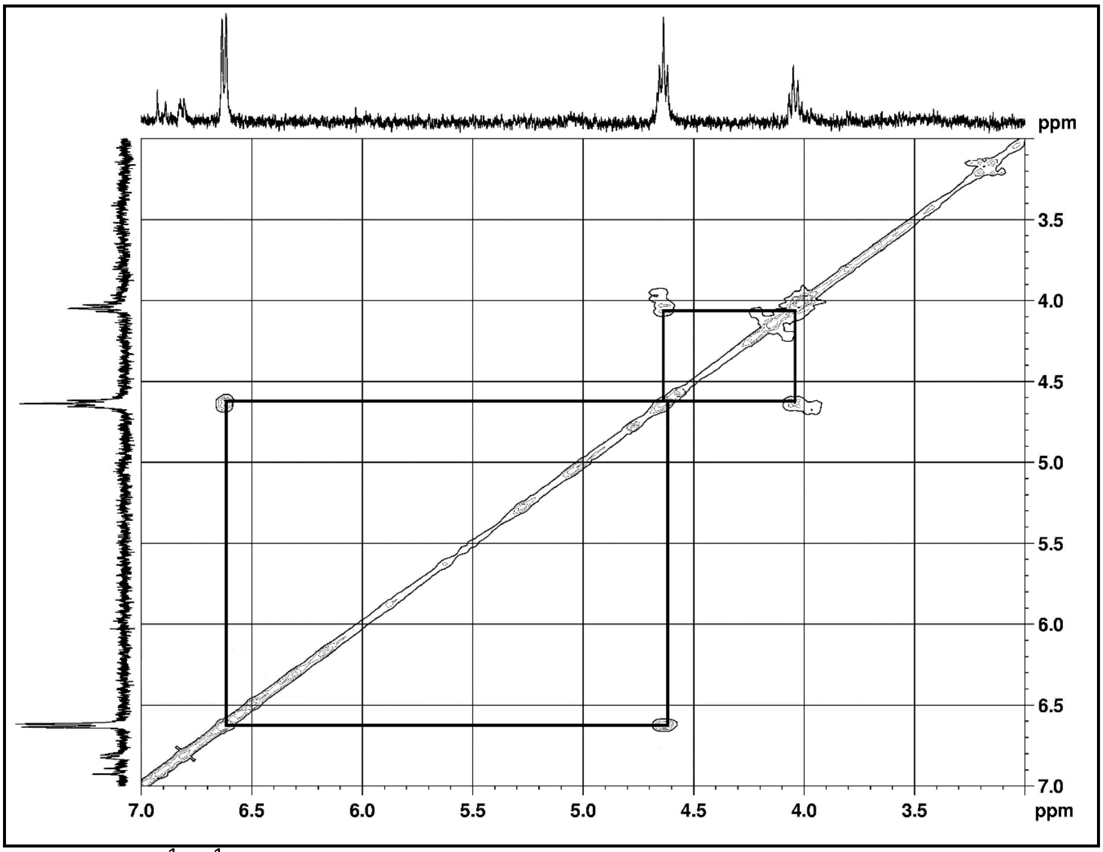

Figure S3. ^{1}H-^{1}H COSY NMR spectrum of **4a** (3.0–7.0 ppm, Solvent: $CDCl_3$)

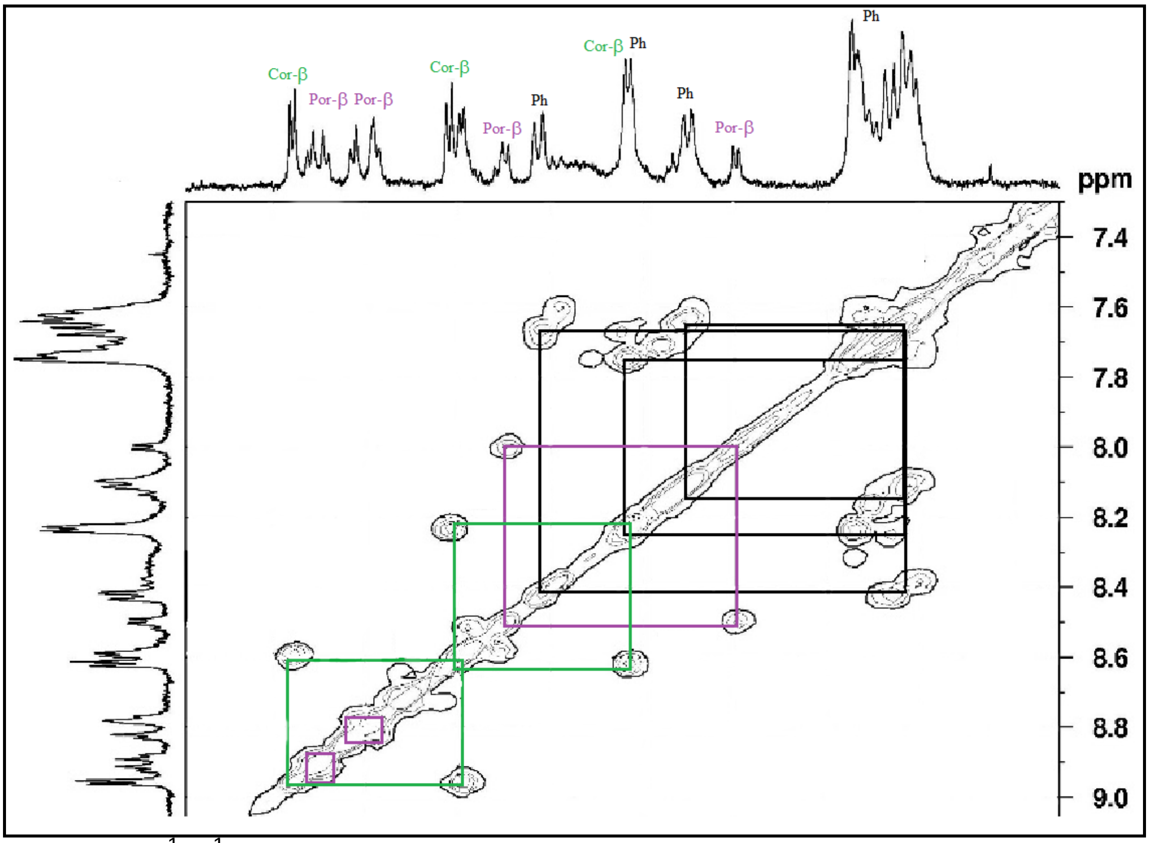

Figure S4. ^{1}H-^{1}H COSY NMR spectrum of **4a** (7.3–9.0 ppm, Solvent: $CDCl_3$)

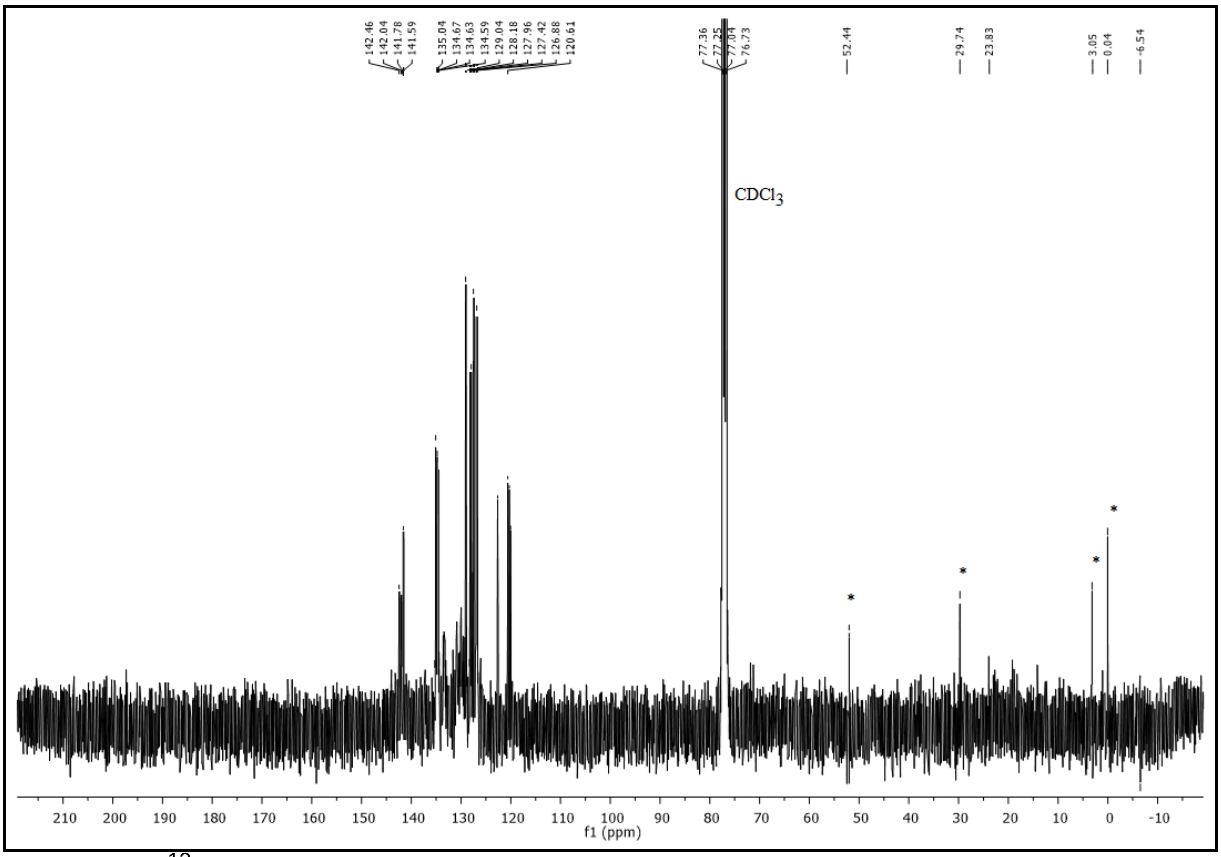

Figure S5. ^{13}C NMR spectrum of **4a** (Solvent: $CDCl_3$) * Solvent impurities

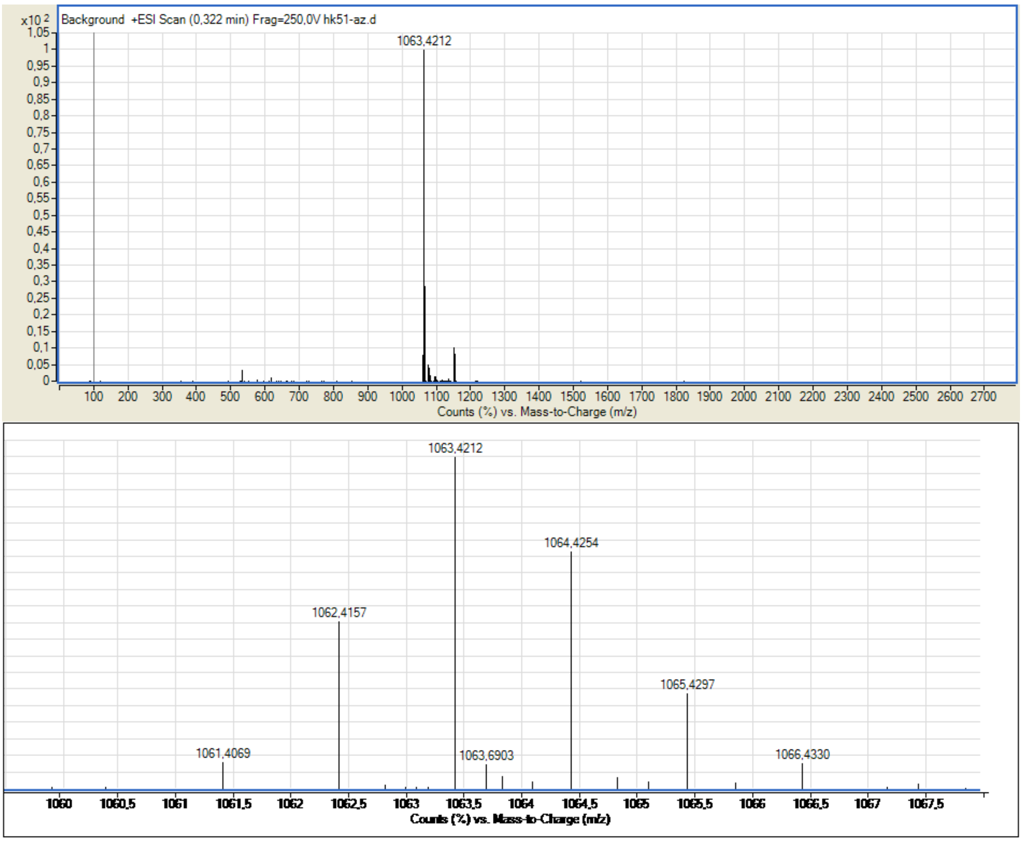

Figure S6. HRMS of **4a**.

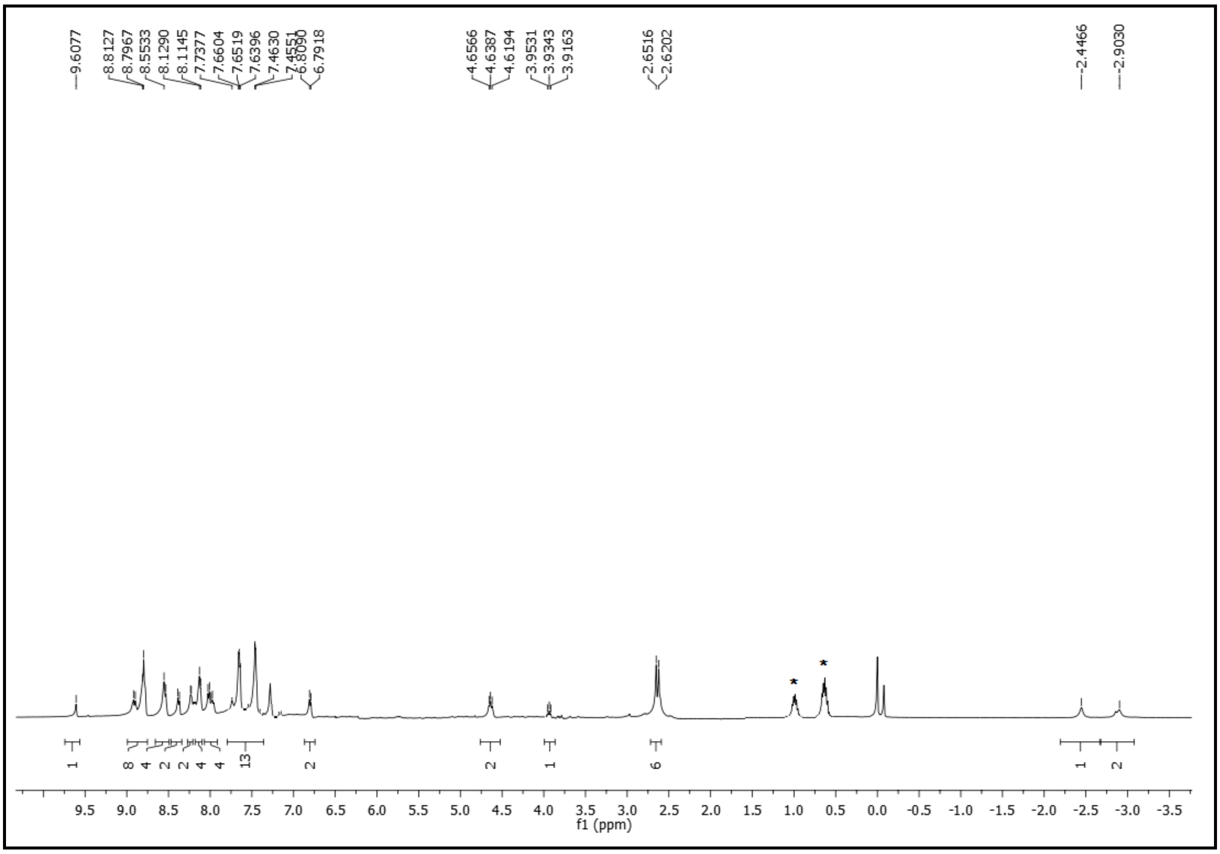

Figure S7. ^{1}H NMR spectrum of **4b** (Solvent: $CDCl_3$) * Solvent impurities

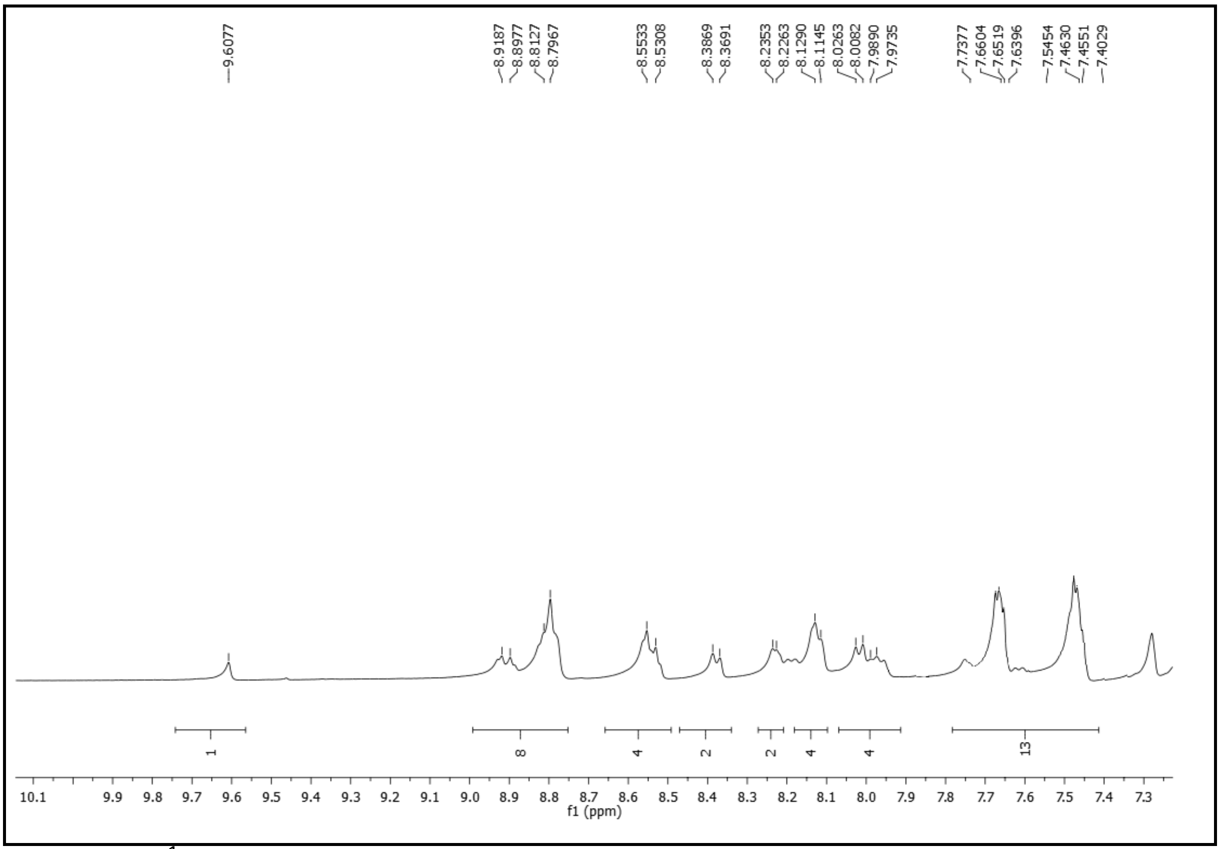

Figure S8. ^{1}H NMR spectrum of **4b** (7.2–10.1 ppm, Solvent: $CDCl_3$)

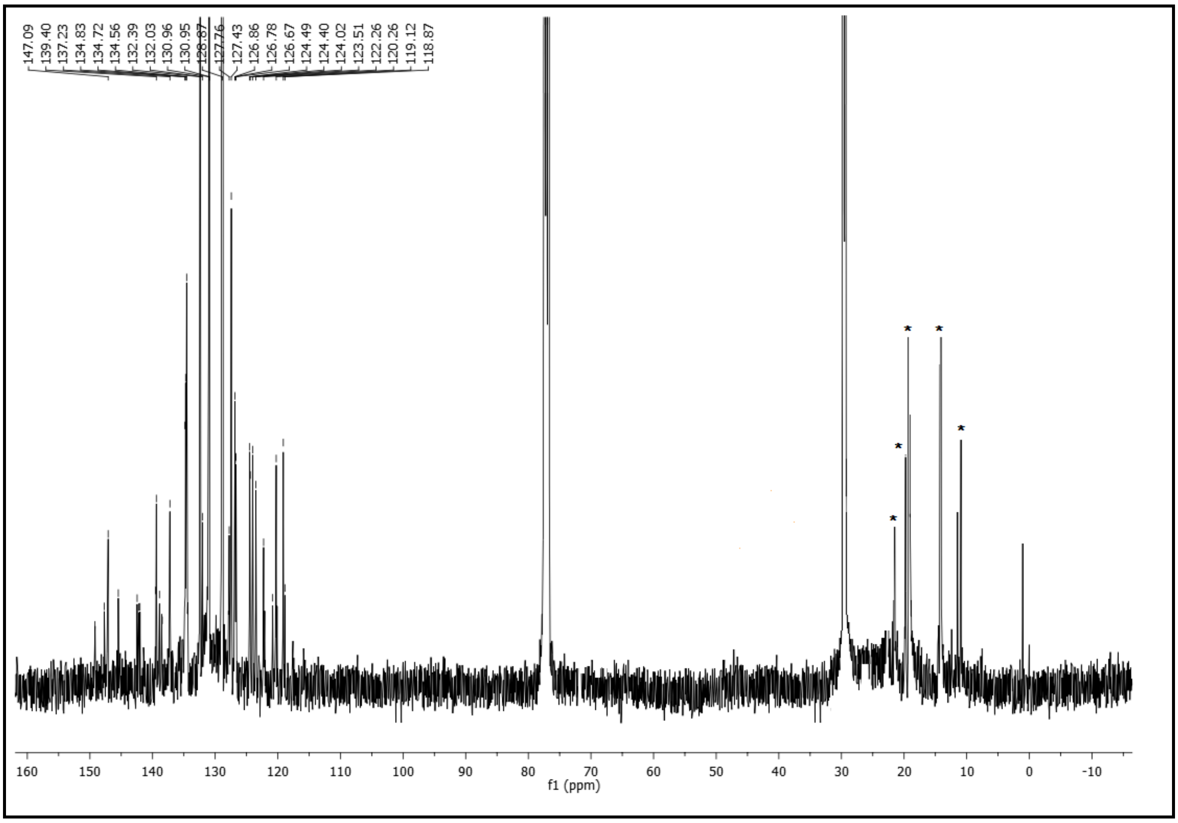

Figure S9. ^{13}C NMR spectrum of **4b** (Solvent: $CDCl_3$) * Solvent impurities

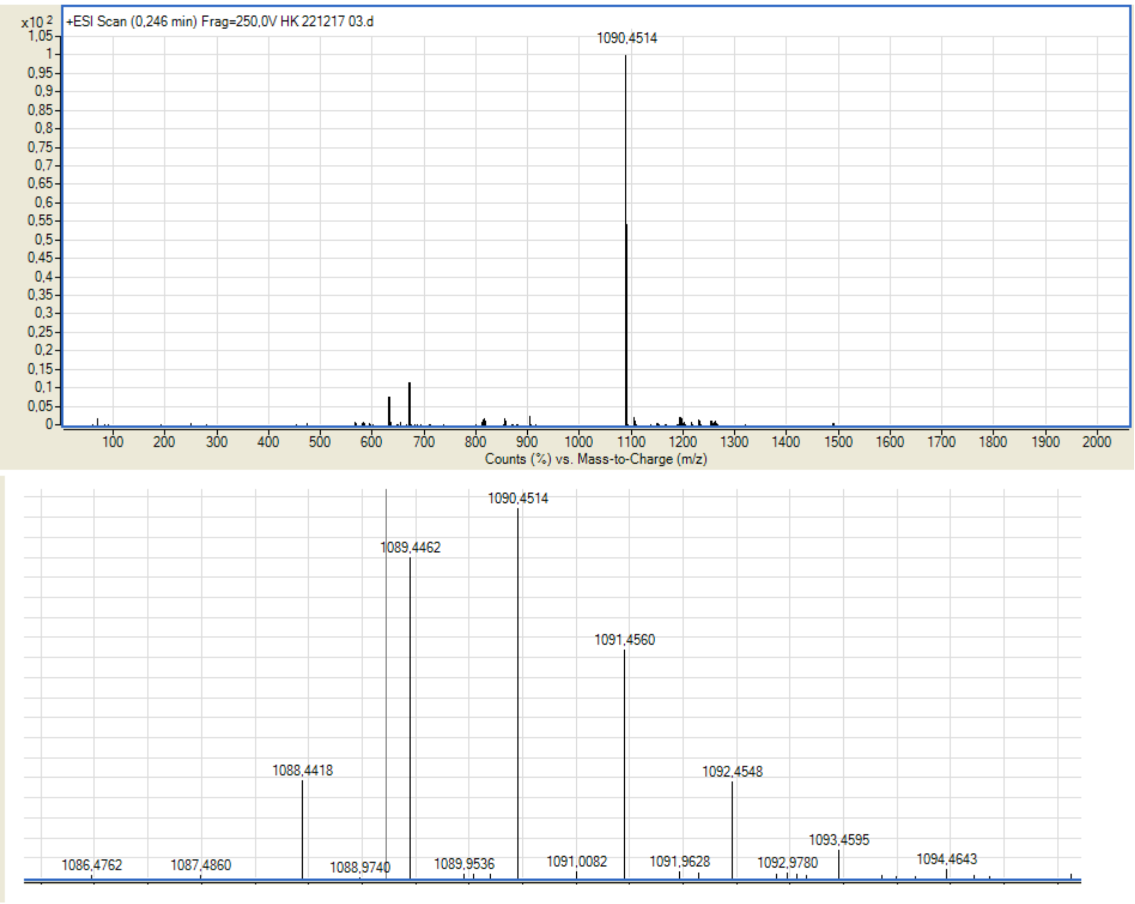

Figure S10. HRMS of **4b**.

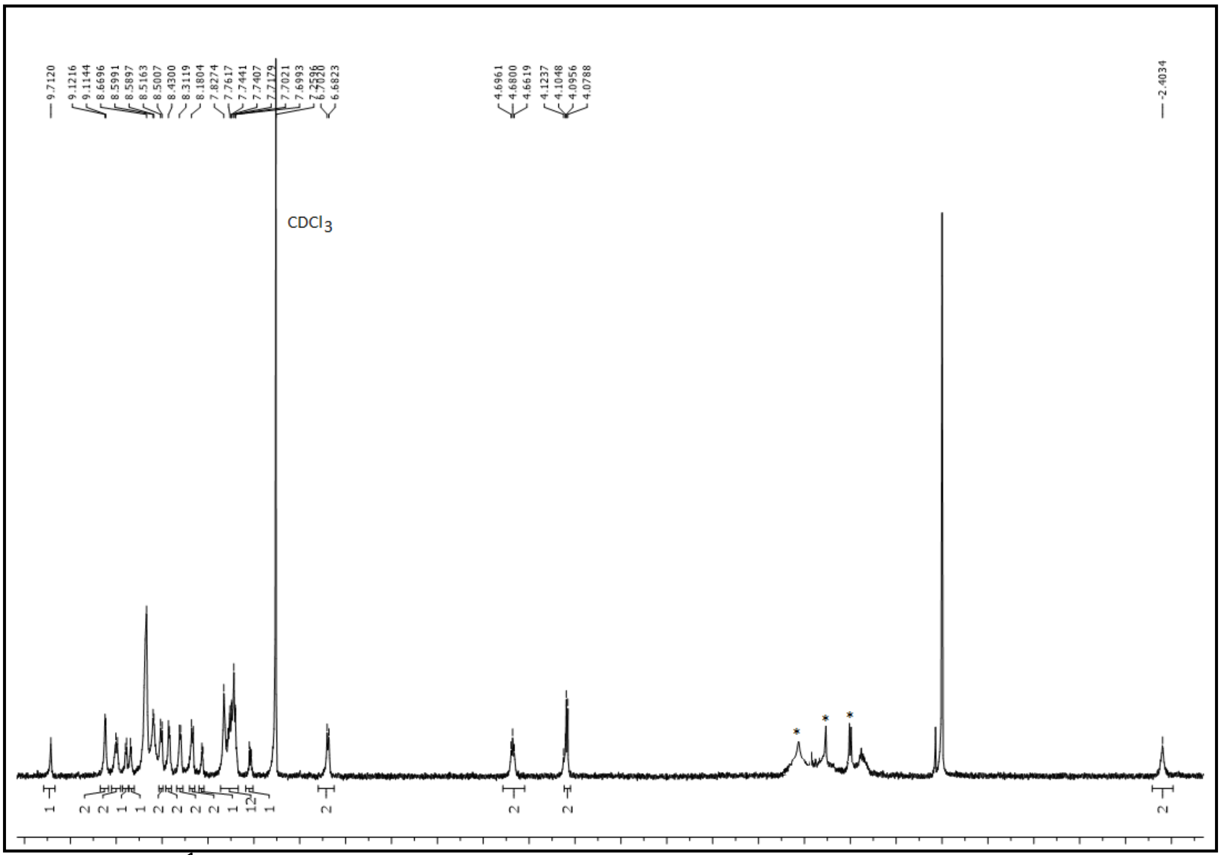

Figure S11. ^{1}H NMR spectrum of **4c** (Solvent: $CDCl_3$) * Solvent impurities

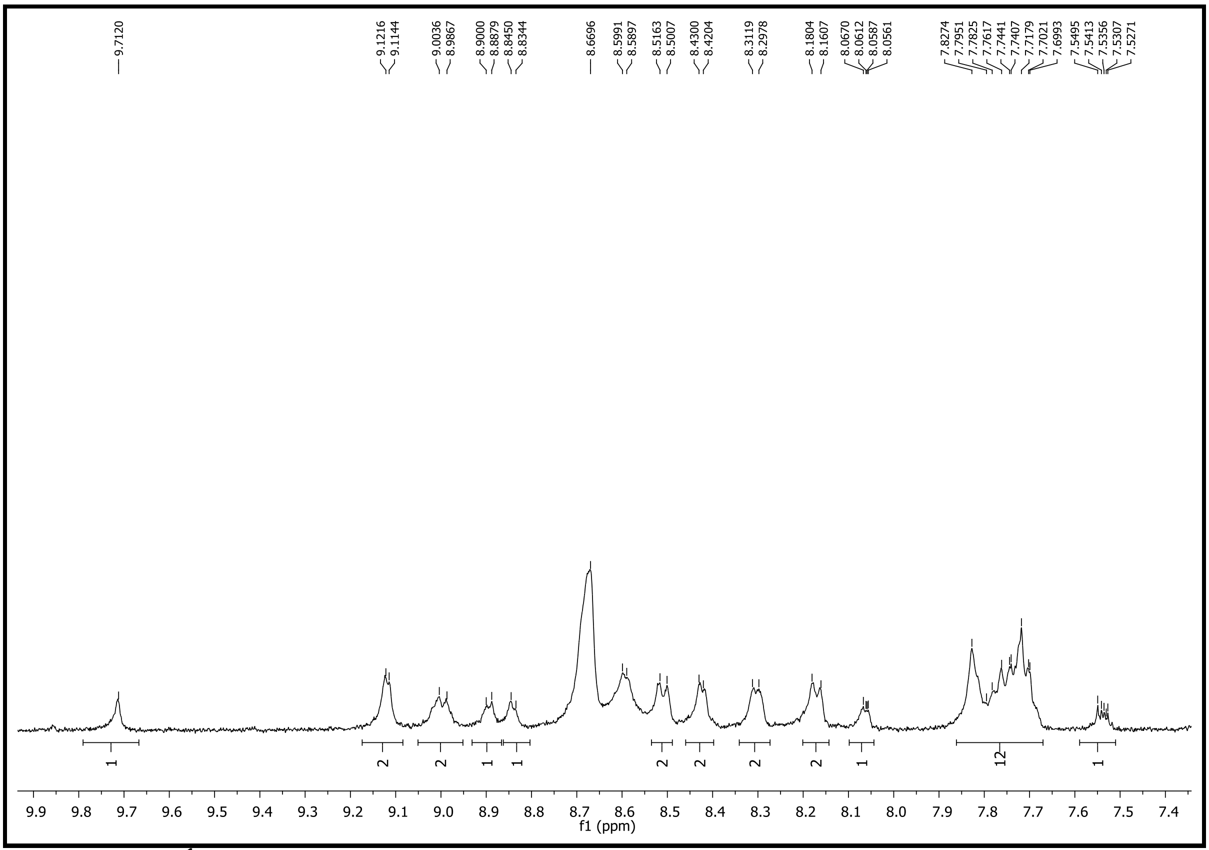

Figure S12. ^{1}H NMR spectrum of **4c** (7.3–9.9 ppm, Solvent: $CDCl_3$)

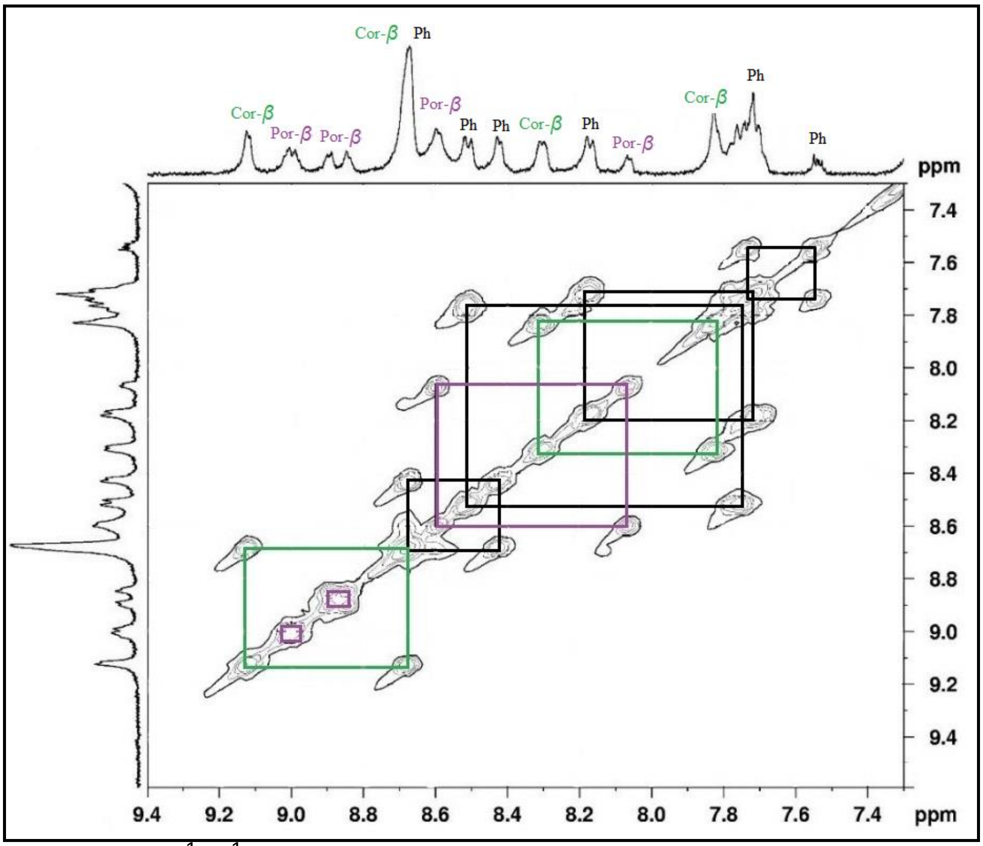

Figure S13. ^{1}H-^{1}H COSY NMR spectrum of **4c** (7.3–9.4 ppm, Solvent: $CDCl_3$)

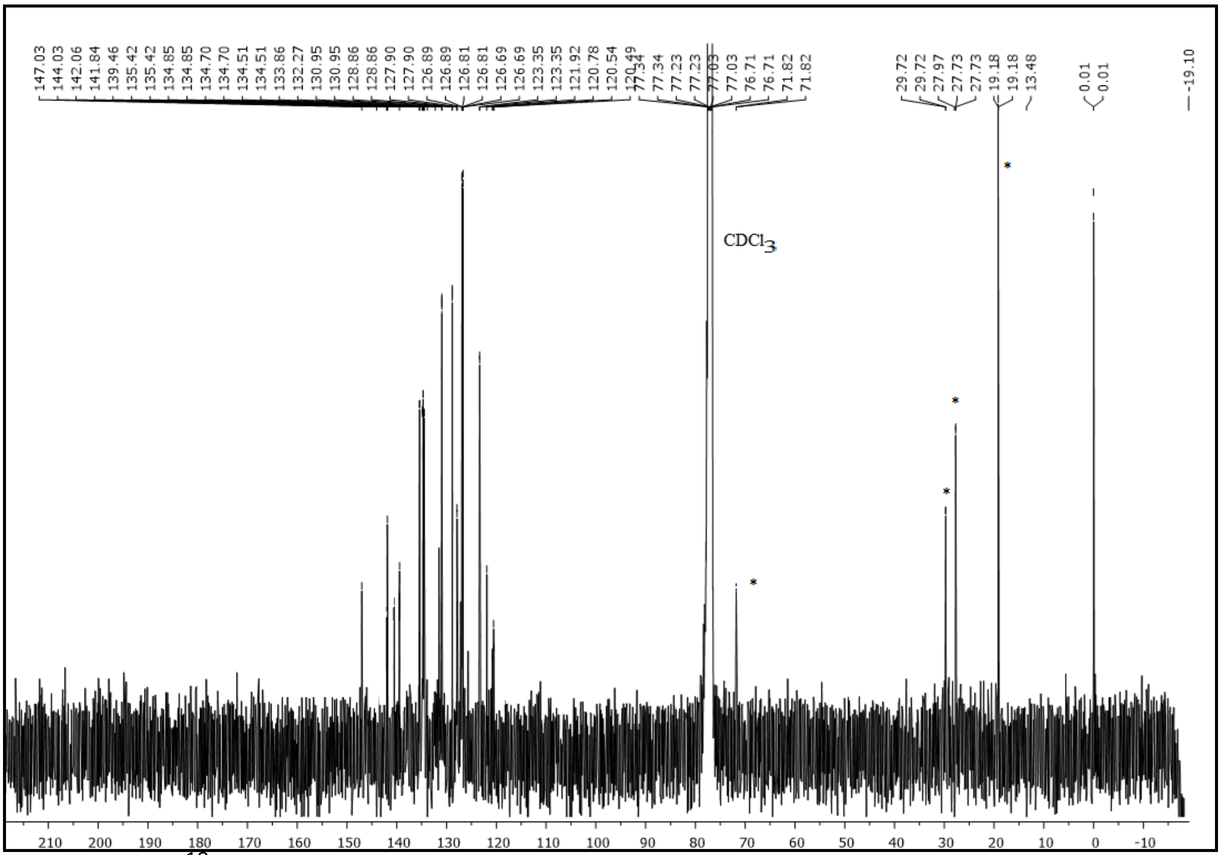

Figure S14. ^{13}C NMR spectrum of **4c** (Solvent: $CDCl_3$) * Solvent impurities

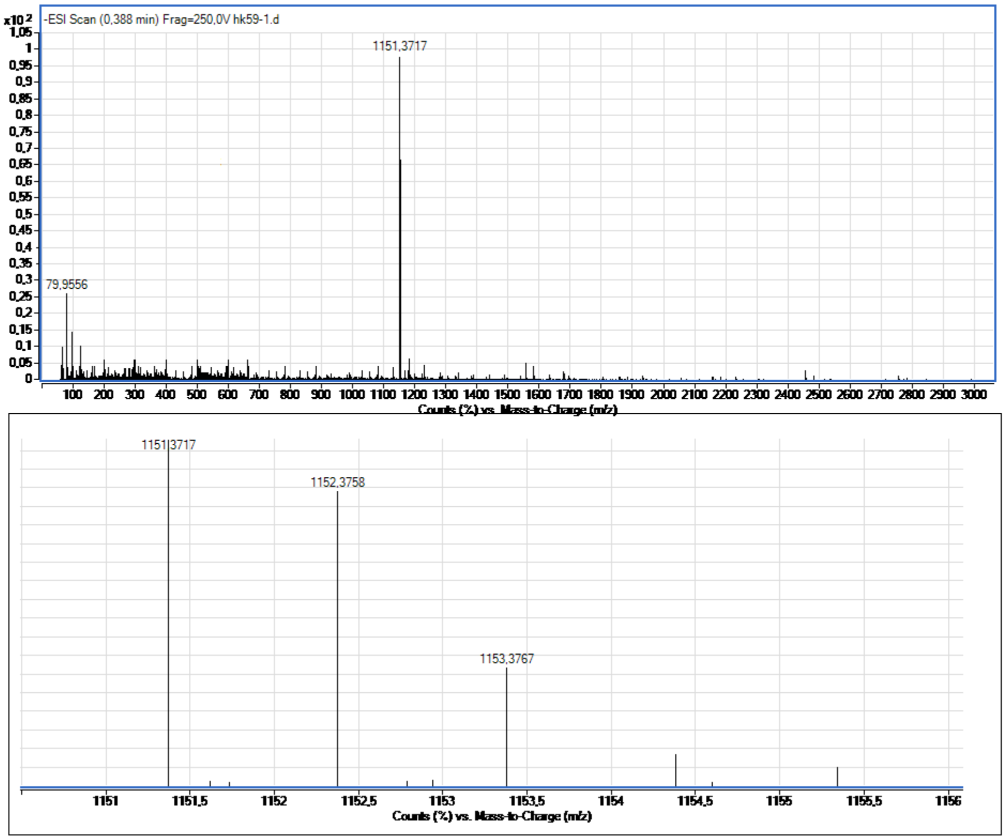

Figure S15. HRMS of **4c**.

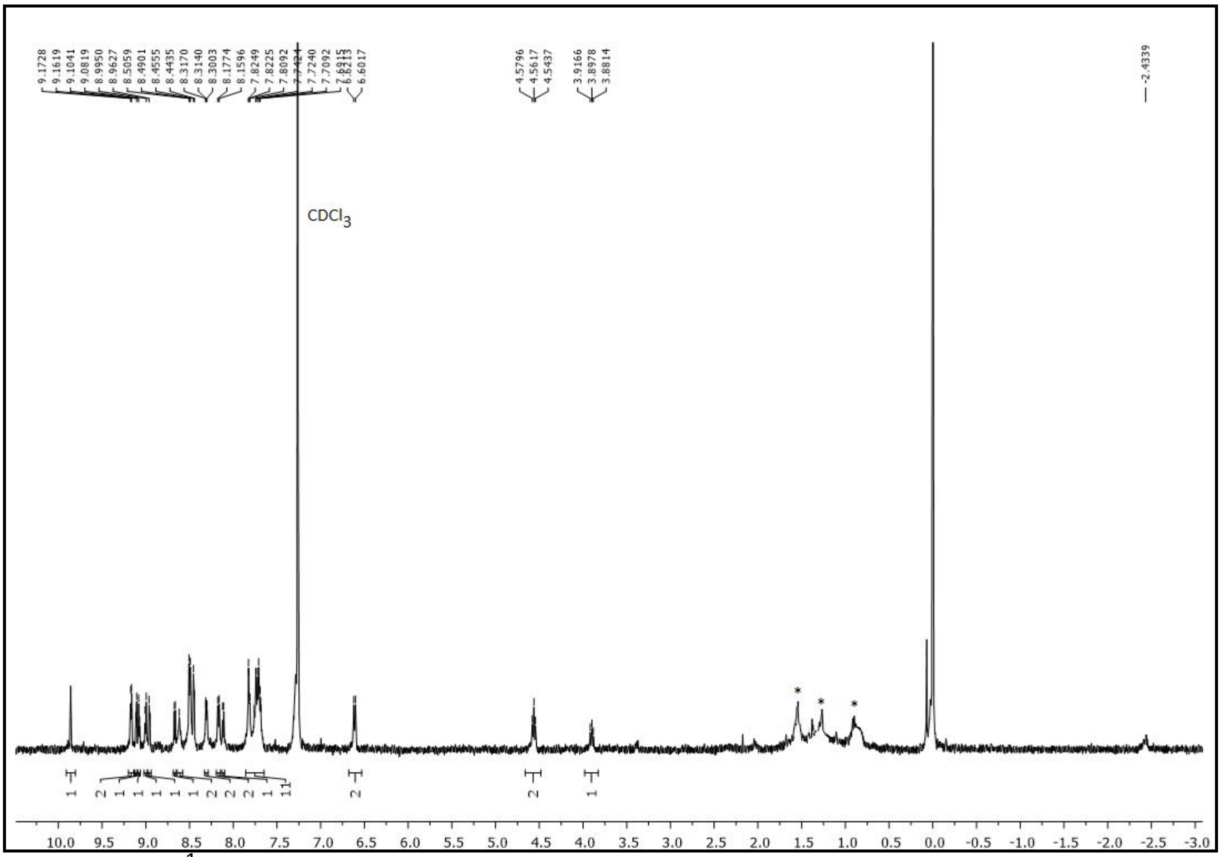

Figure S16. ^{1}H NMR spectrum of **4e** (Solvent: $CDCl_3$) * Solvent impurities

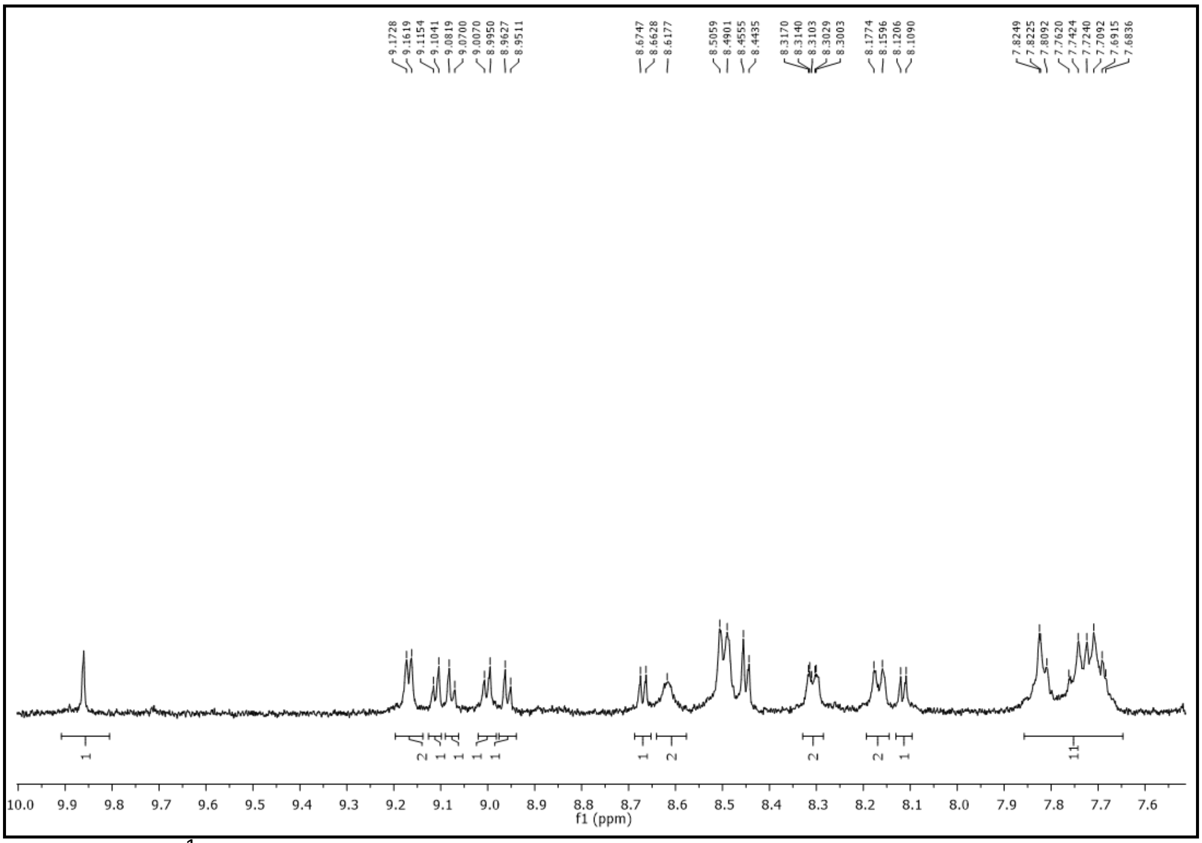

Figure S17. ^{1}H NMR spectrum of **4e** (7.5–10.0 ppm, Solvent: $CDCl_3$)

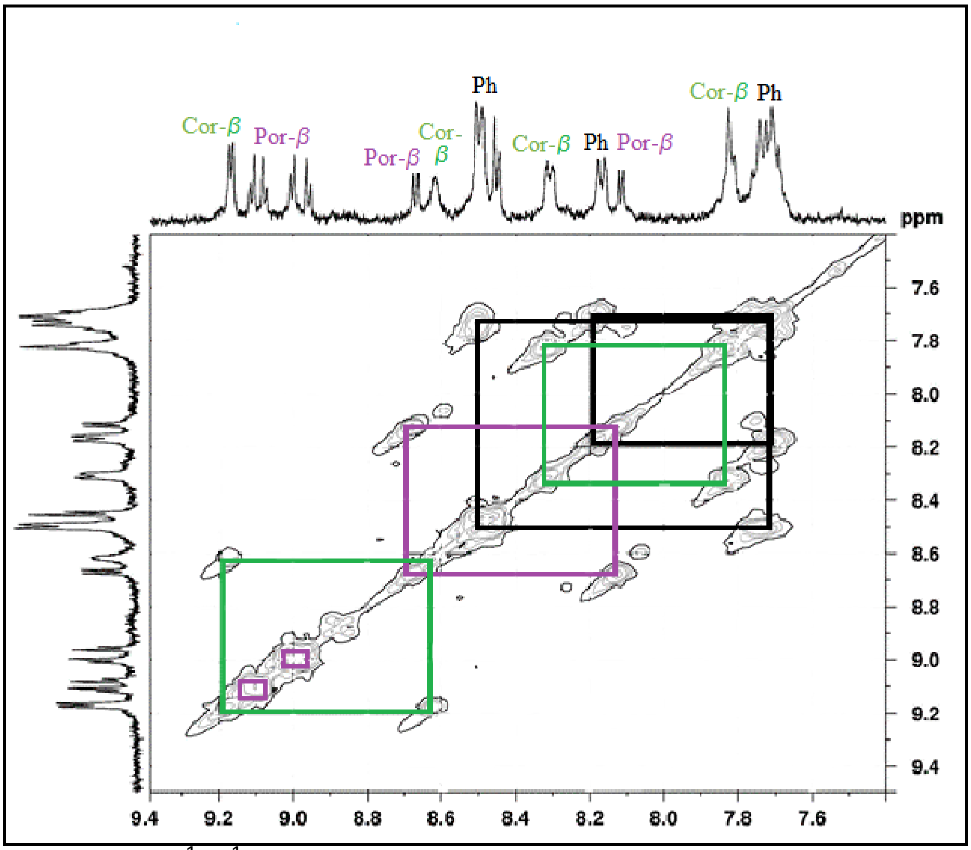

Figure S18. ^{1}H-^{1}H COSY NMR spectrum of **4e** (7.5–9.4 ppm, Solvent: $CDCl_3$)

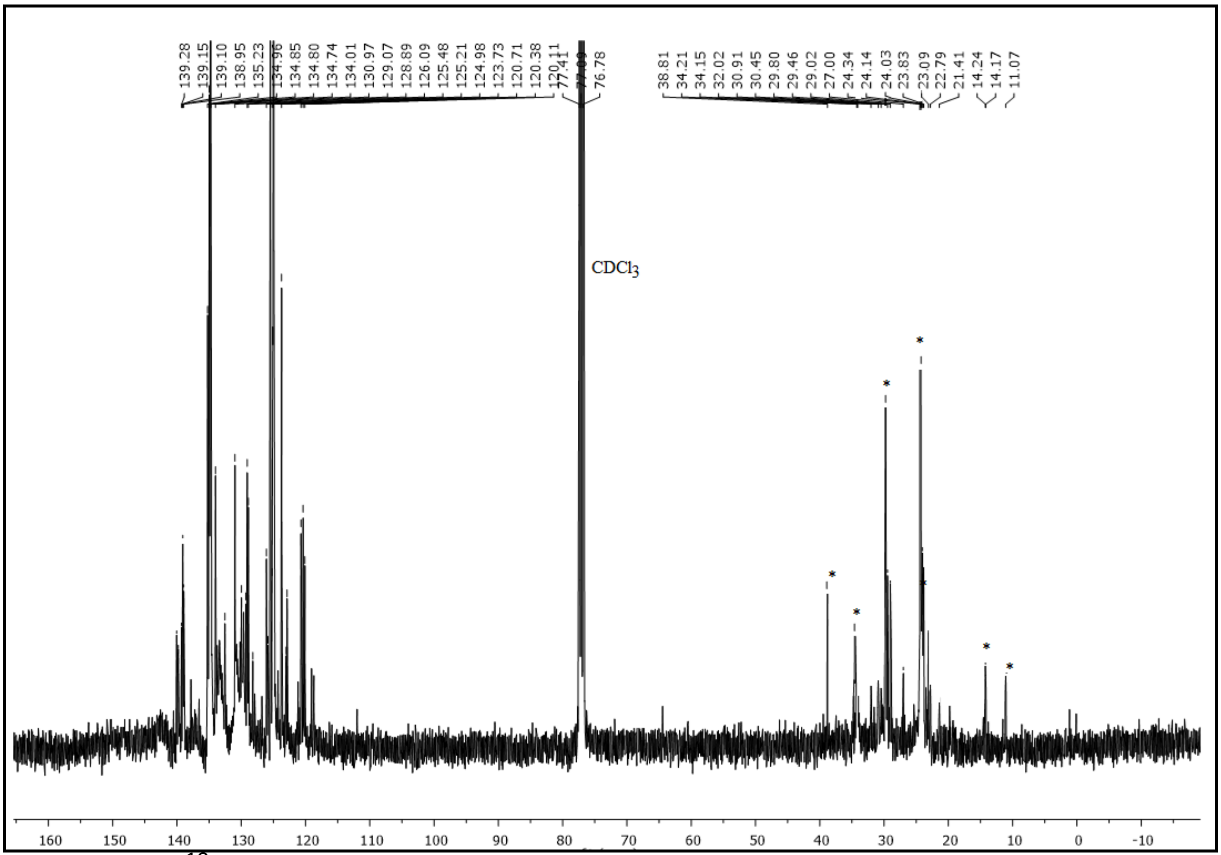

Figure S19. ^{13}C NMR spectrum of **4e** (Solvent: $CDCl_3$) * Solvent impurities

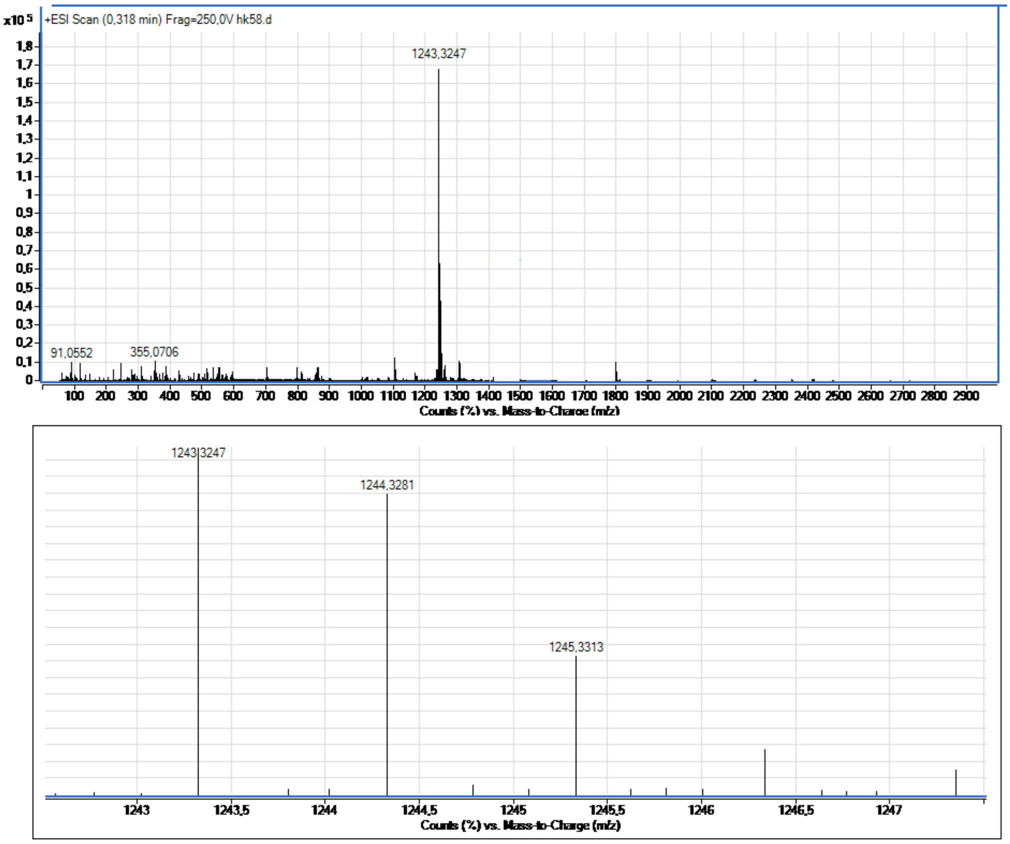

Figure S20. HRMS of **4e**.

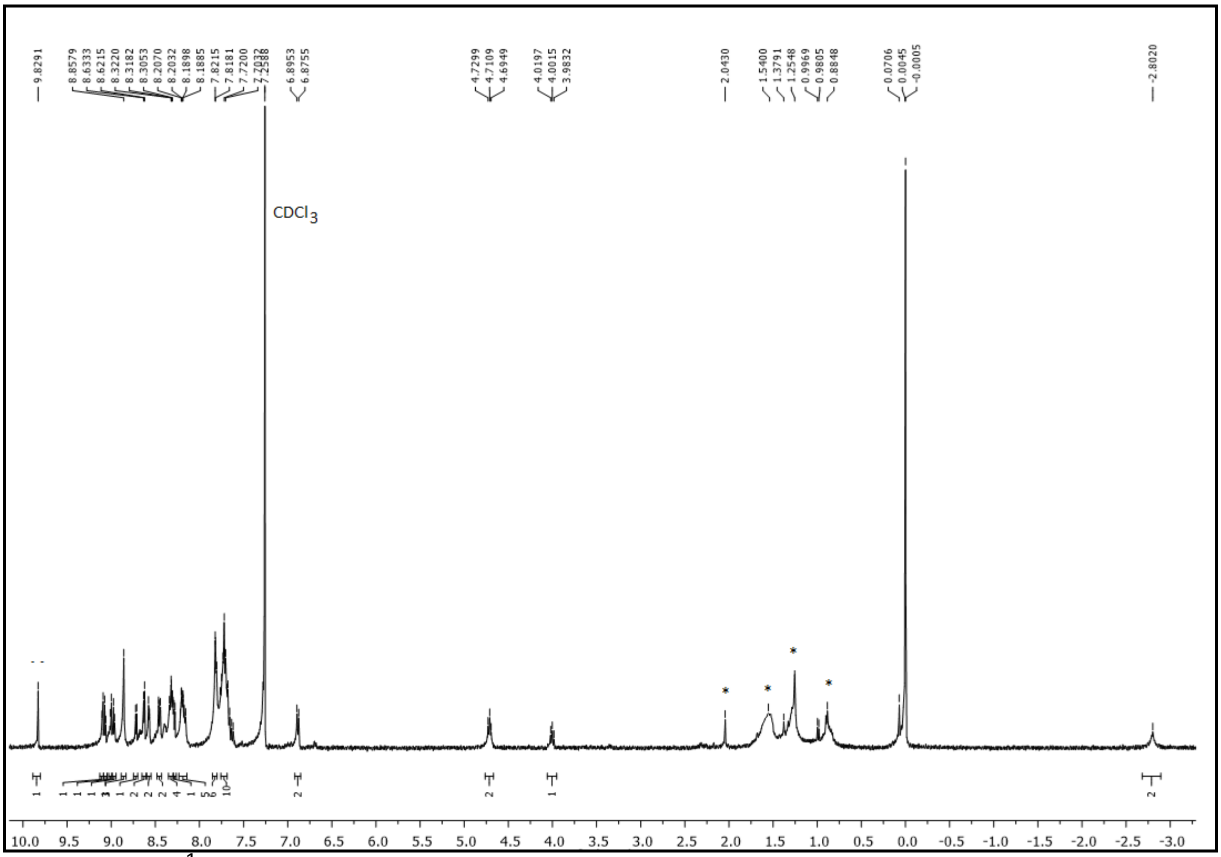

Figure S21. ^{1}H NMR spectrum of **4f** (Solvent: $CDCl_3$) * Solvent impurities

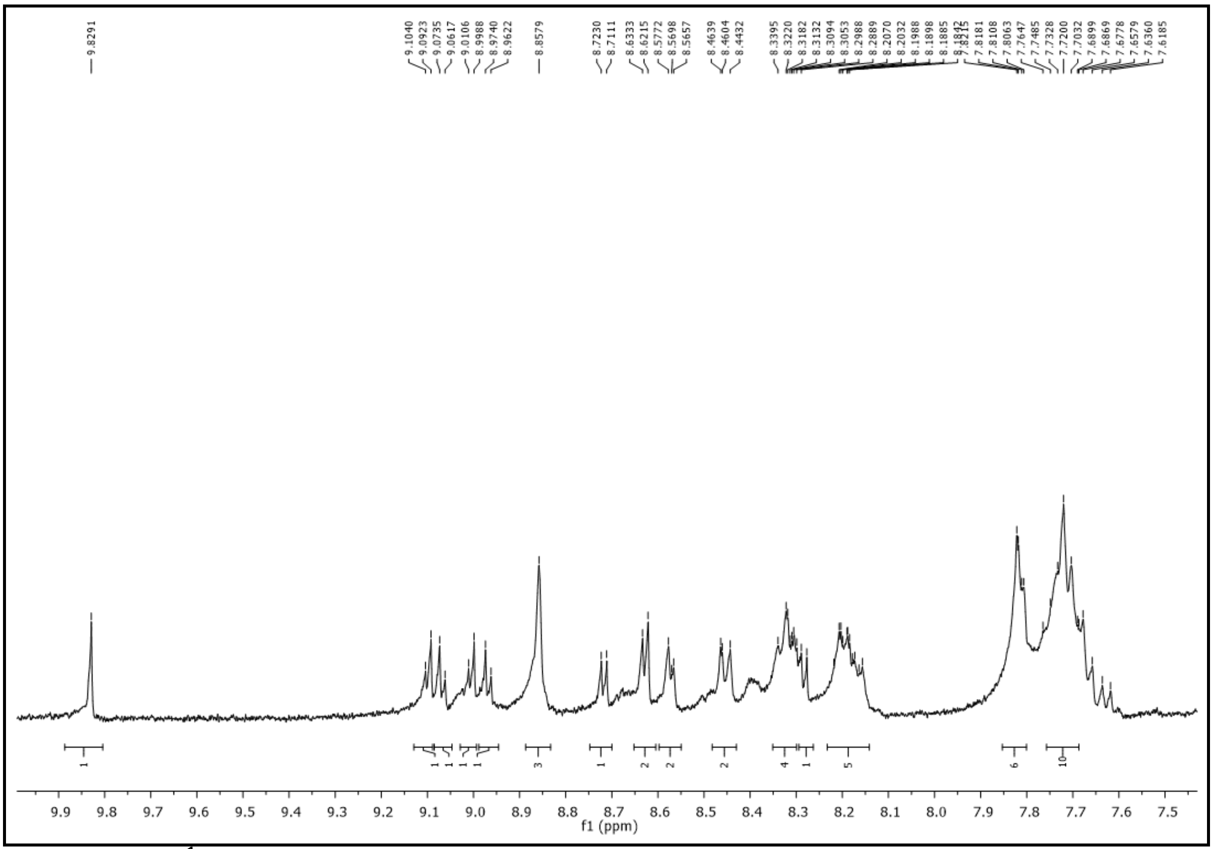

Figure S22. ^{1}H NMR spectrum of **4f** (7.4–10.0 ppm, Solvent: $CDCl_3$)

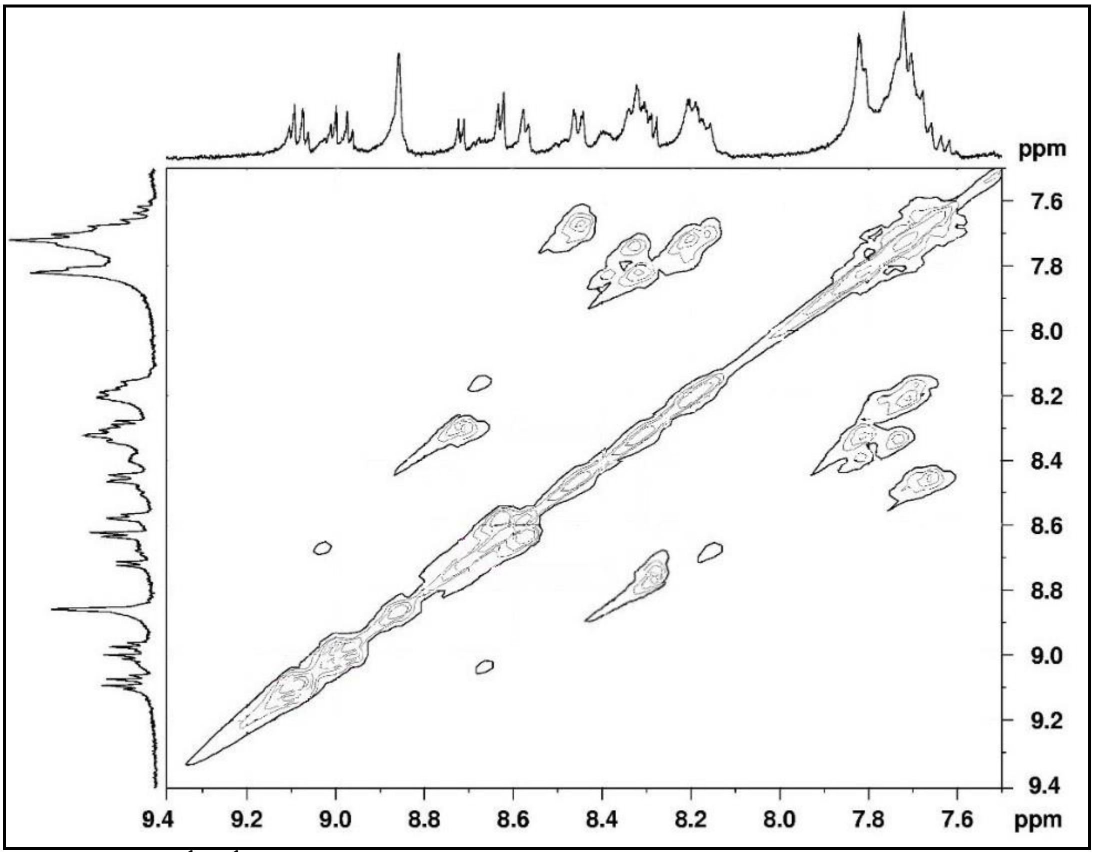

Figure S23. ^{1}H-^{1}H COSY NMR spectrum of **4f** (7.5–9.4 ppm, Solvent: $CDCl_3$)

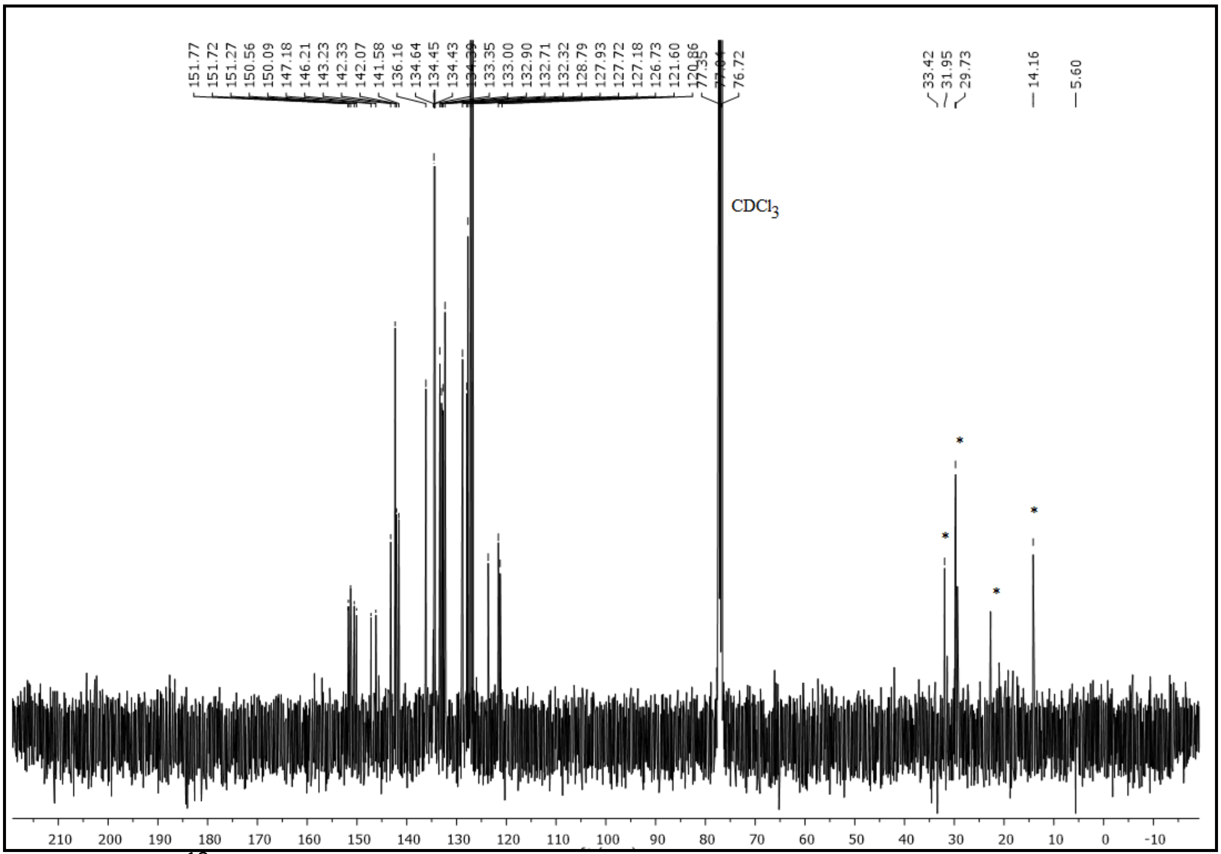

Figure S24. ^{13}C NMR spectrum of **4f** (Solvent: $CDCl_3$) * Solvent impurities

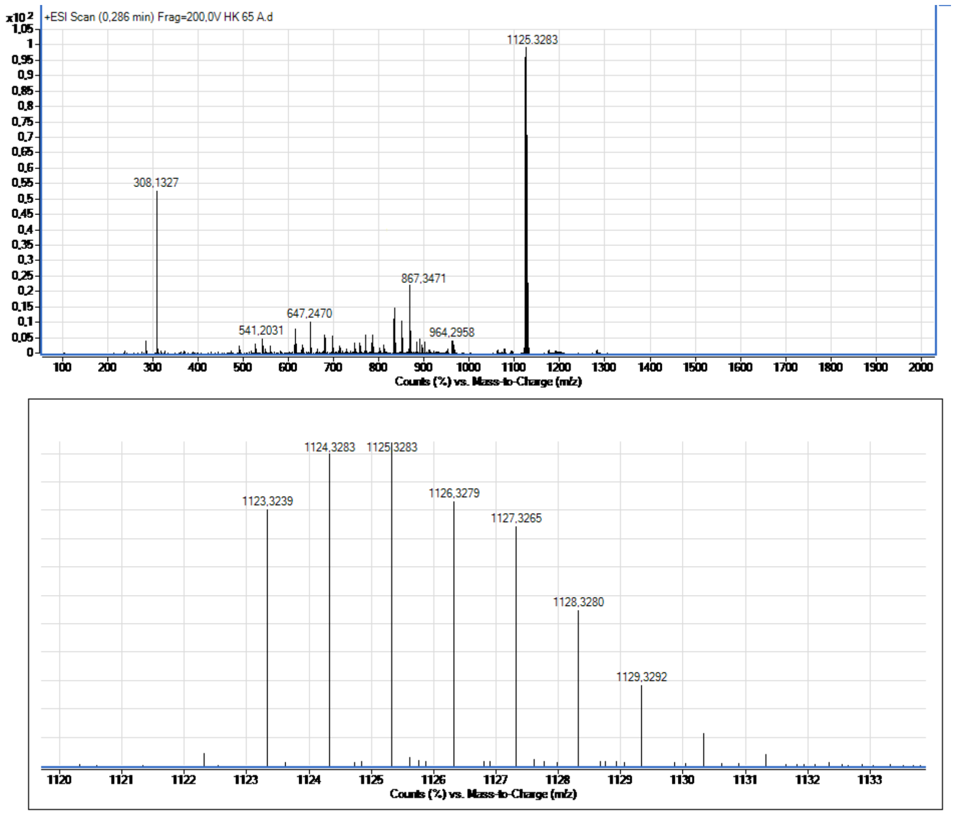

Figure S25. HRMS of **4f**.

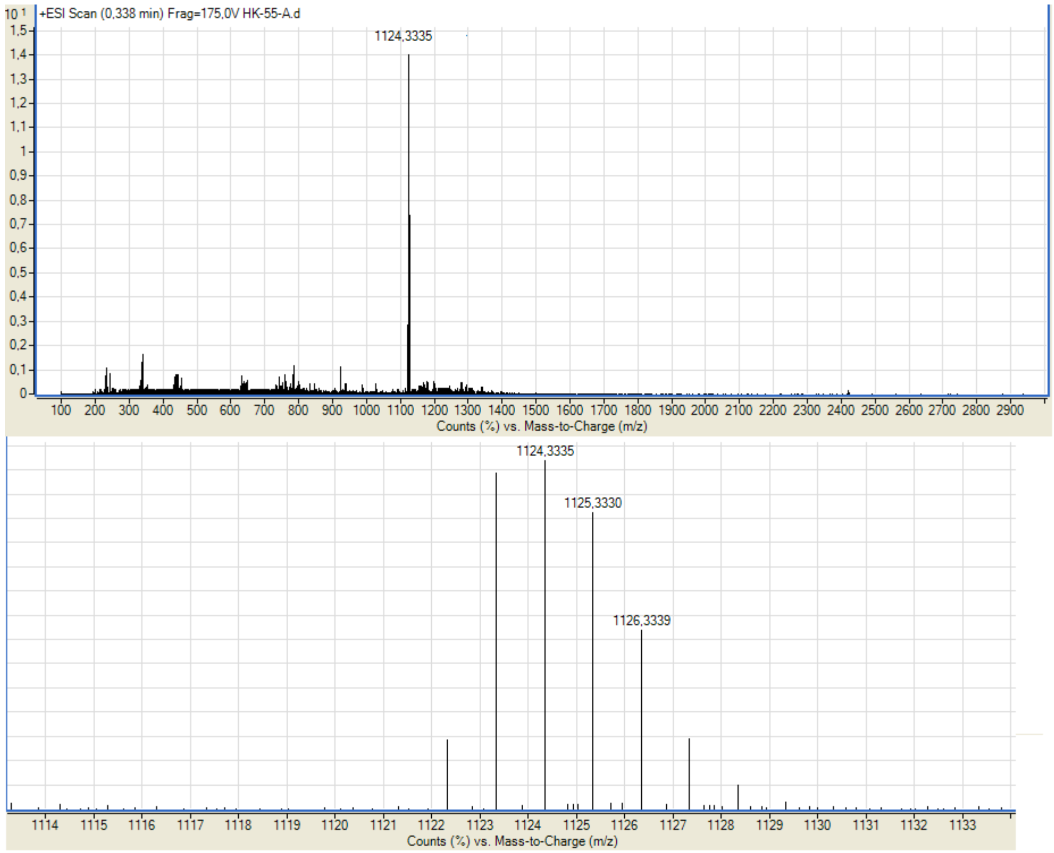

Figure S26. HRMS of **4g**.

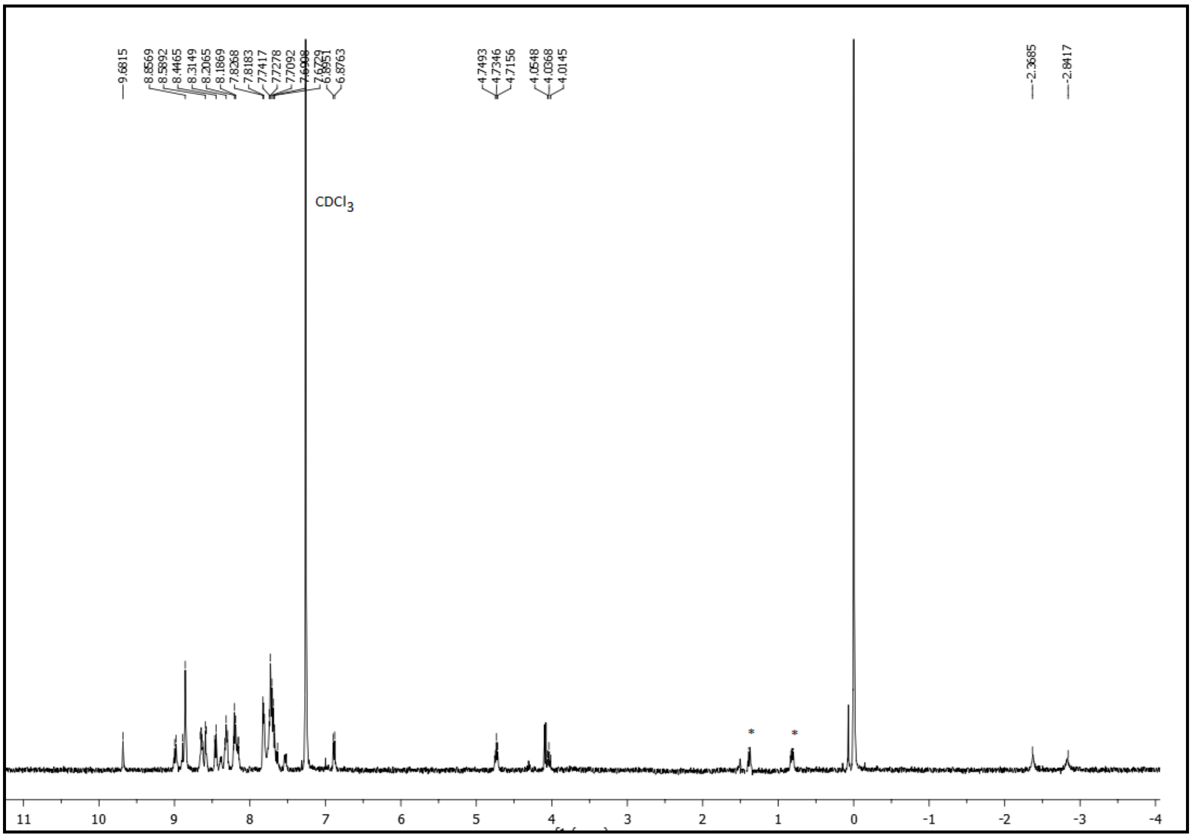

Figure S27. ^{1}H NMR spectrum of **5a** * Solvent impurities

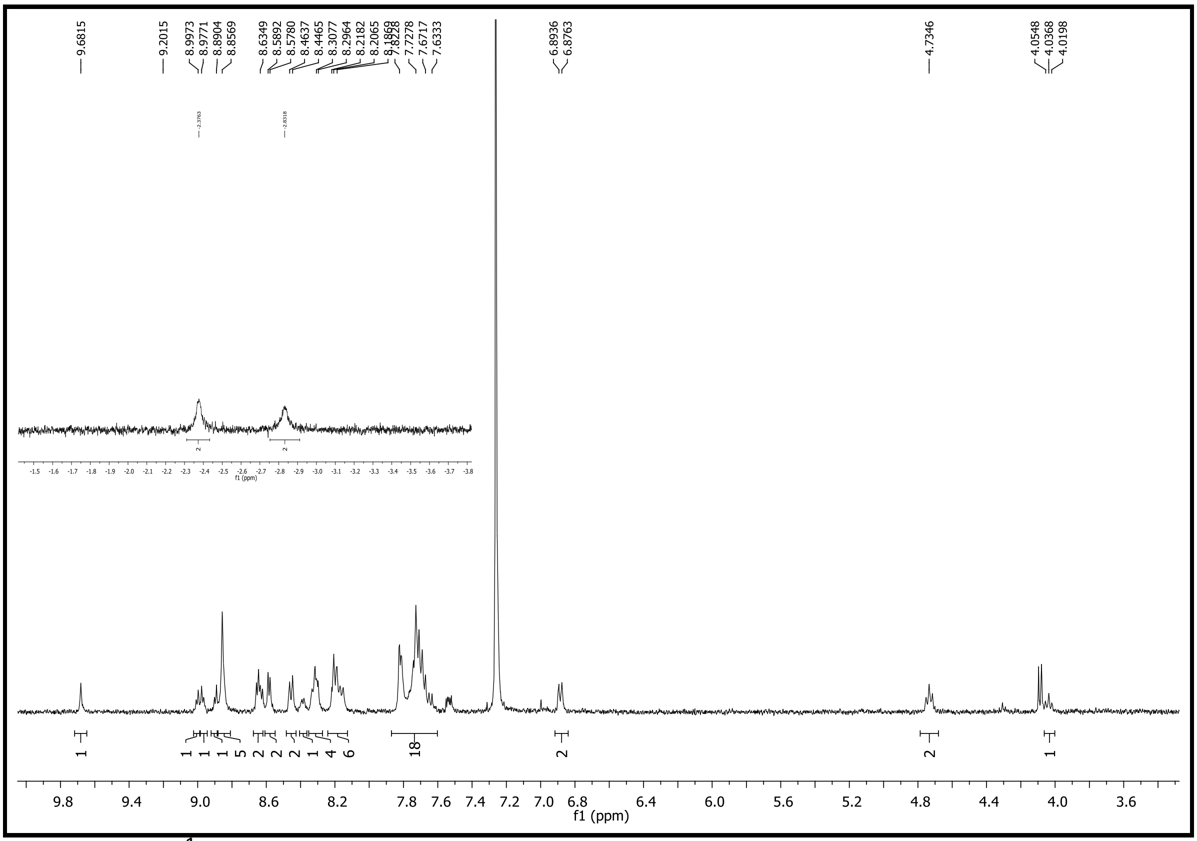

Figure S28. ^{1}H NMR spectrum of **5a** (Solvent: $CDCl_3$)

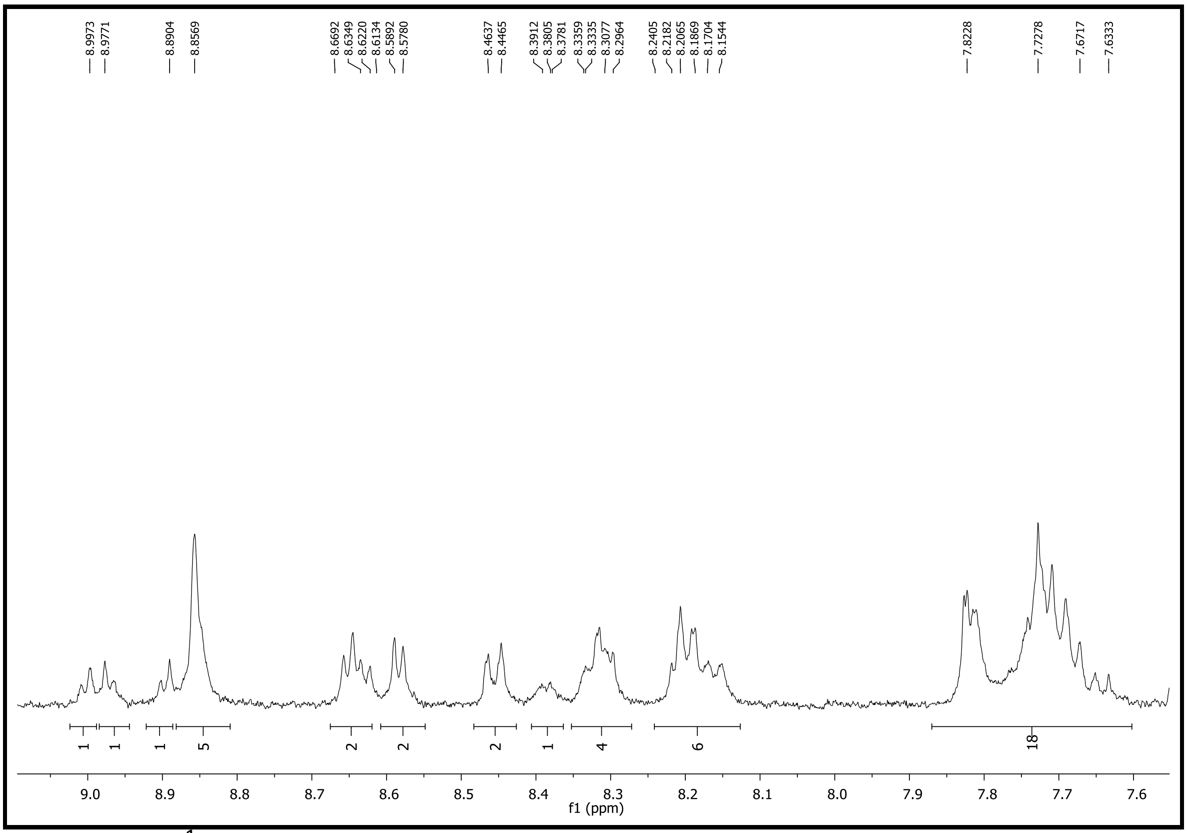

Figure S29. ^{1}H NMR spectrum of **5a** (7.5–9.2 ppm, Solvent: $CDCl_3$)

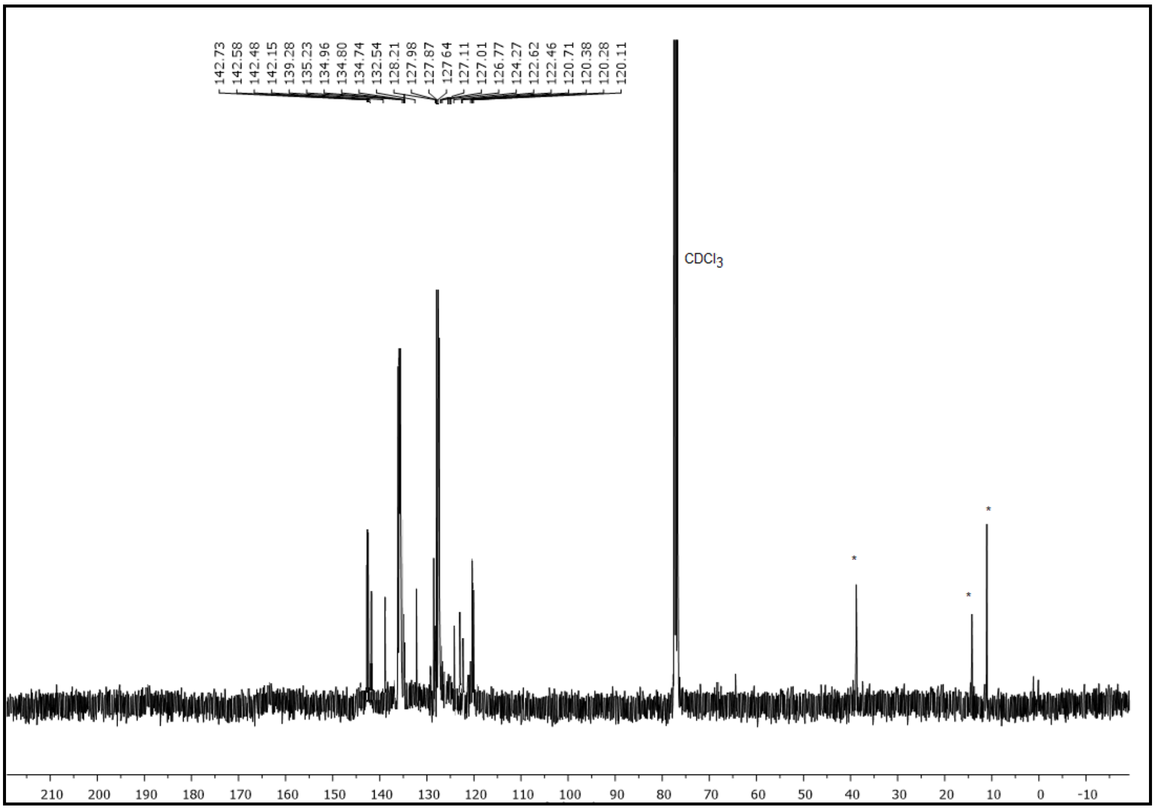

Figure S30. ^{13}C NMR spectrum of **5a** (Solvent: $CDCl_3$) * Solvent impurities

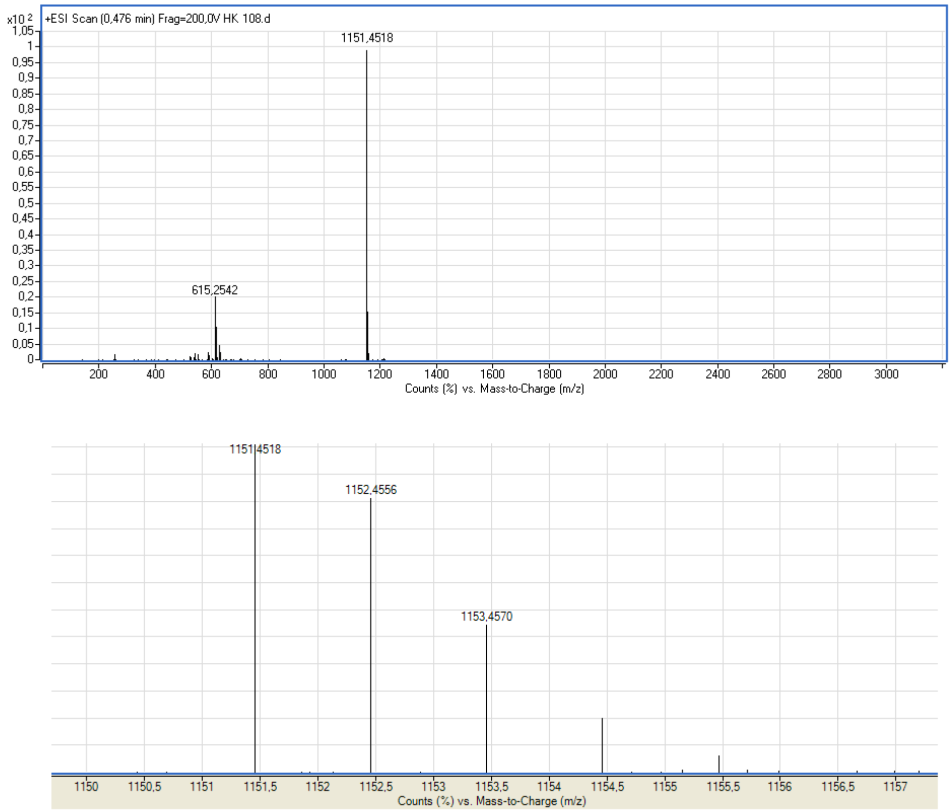

Figure S31. HRMS of **5a**.

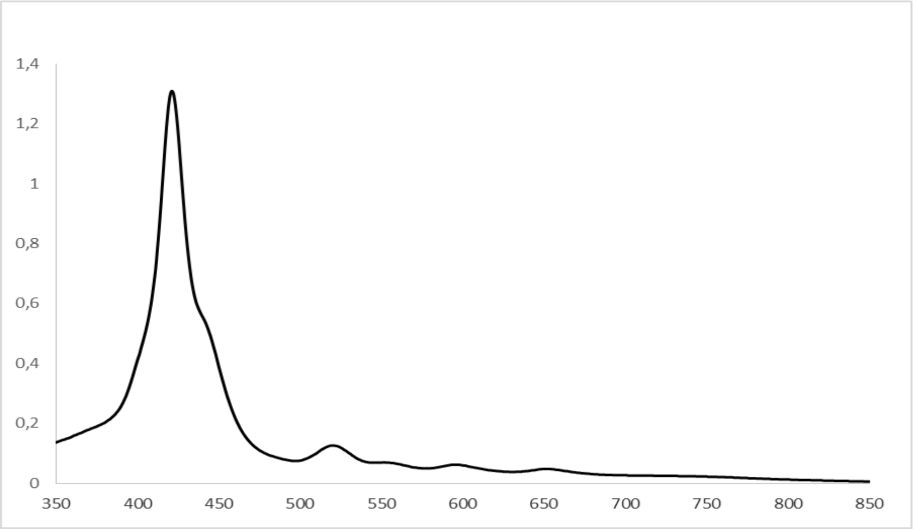

Figure S32. UV–vis absorption spectrum of **4a**.

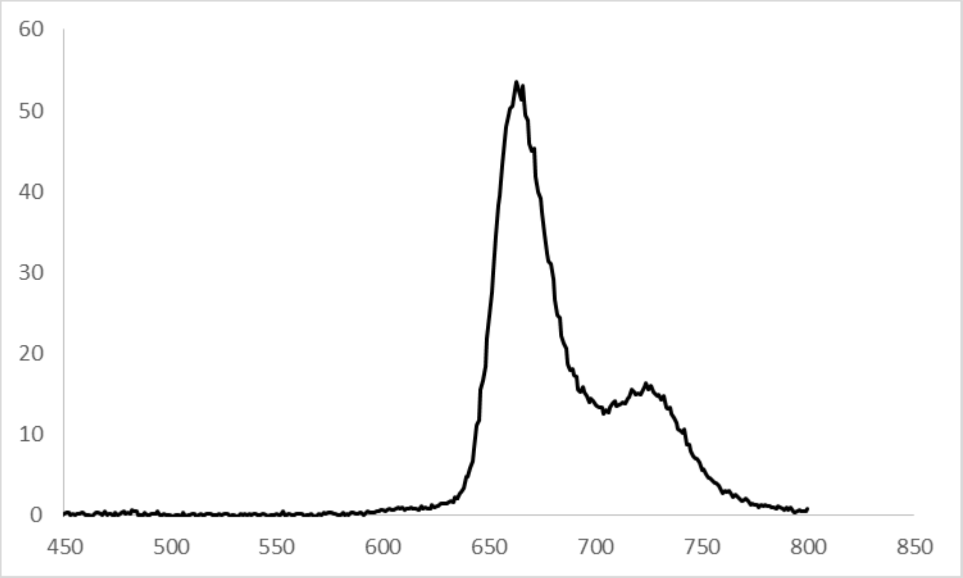

Figure S33. Emission spectrum of **4a** at room temperature (excitation at λ = 420 nm)

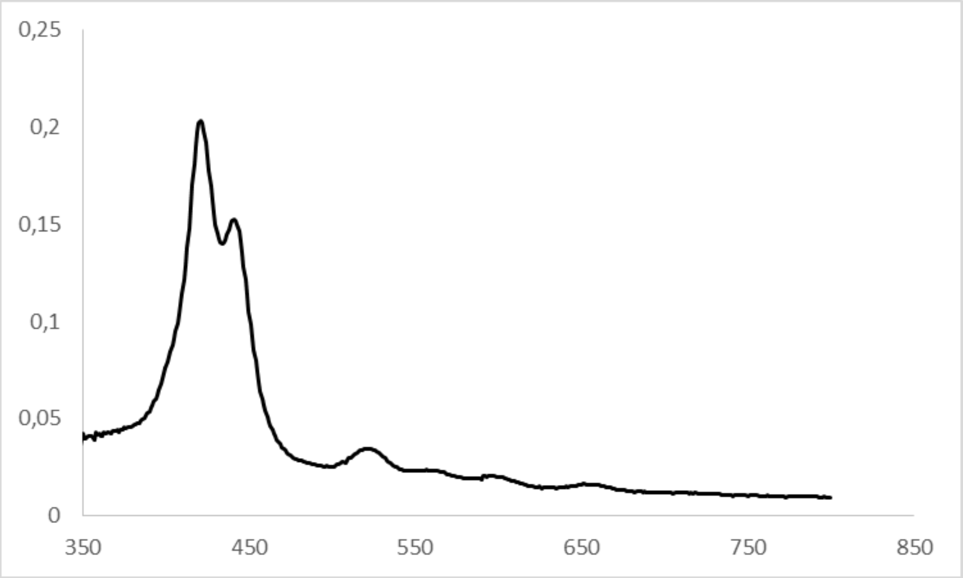

Figure S34. UV–vis absorption spectrum of **4b**.

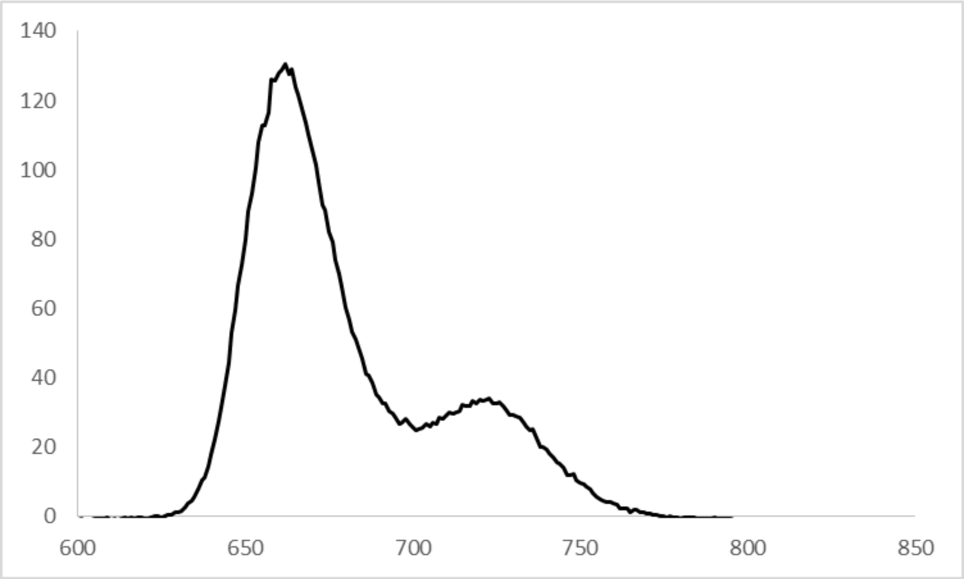

Figure S35. Emission spectrum of **4b** at room temperature (excitation at λ = 420 nm)

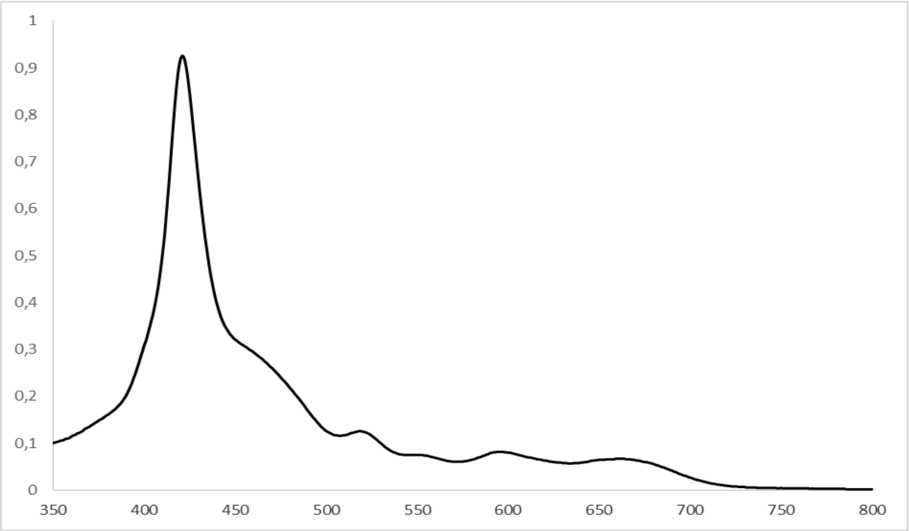

Figure S36. UV–vis absorption spectrum of **4c**.

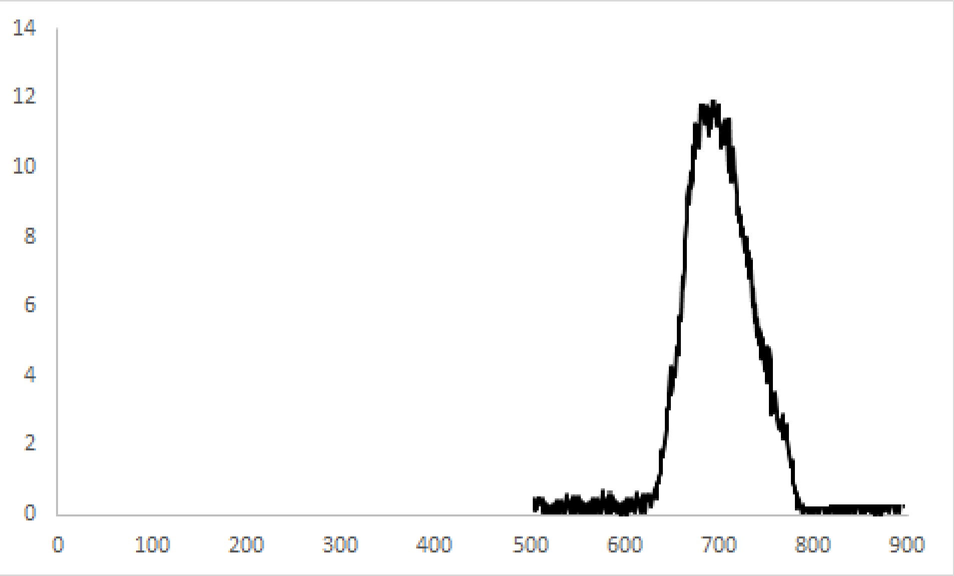

Figure S37. Emission spectrum of **4c** at room temperature (excitation at λ = 420 nm)

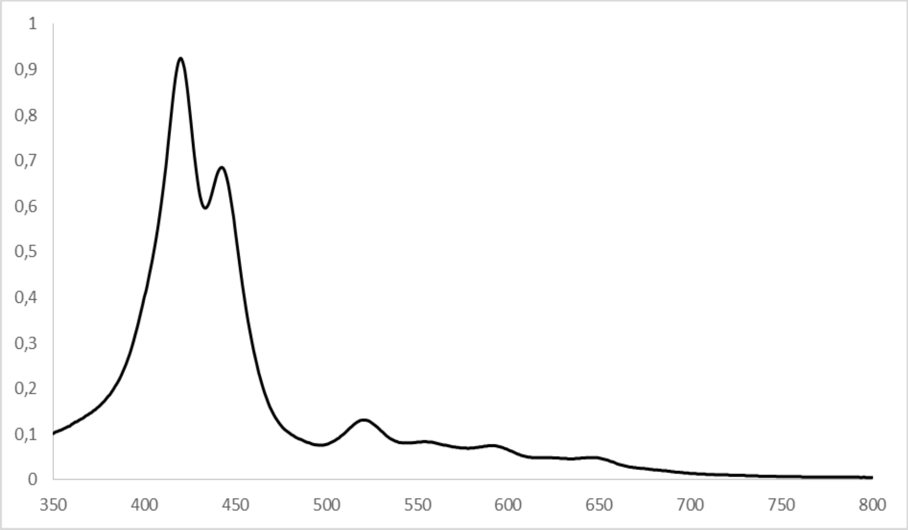

Figure S38. UV–vis absorption spectrum of **4e**.

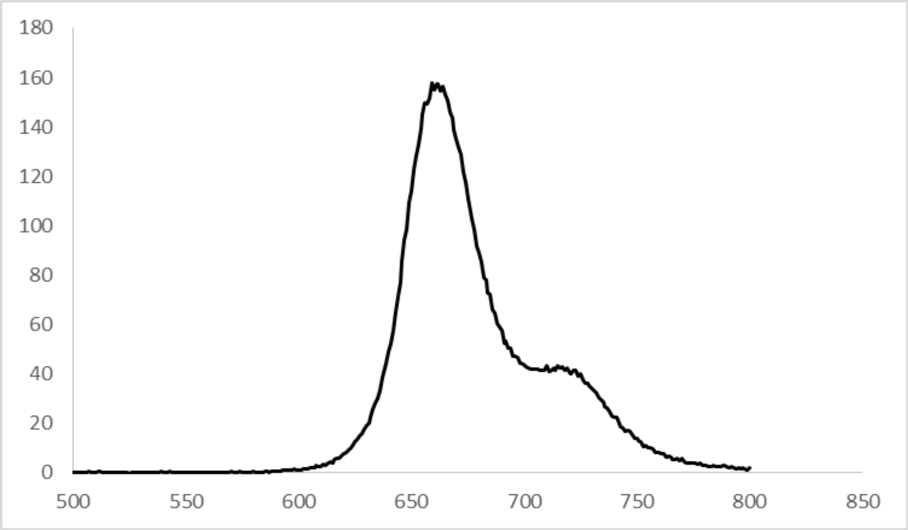

Figure S39. Emission spectrum of **4e** at room temperature (excitation at λ = 420 nm)

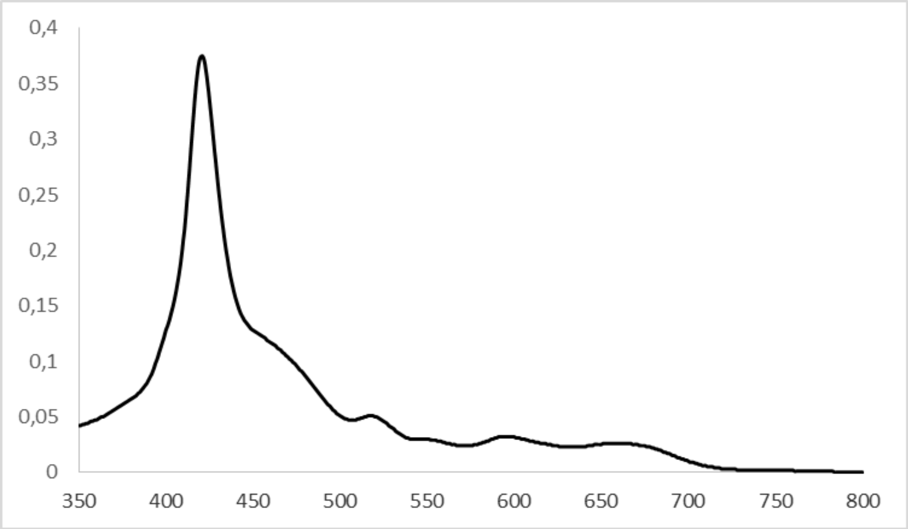

Figure S40 UV–vis absorption spectrum of **4f**.

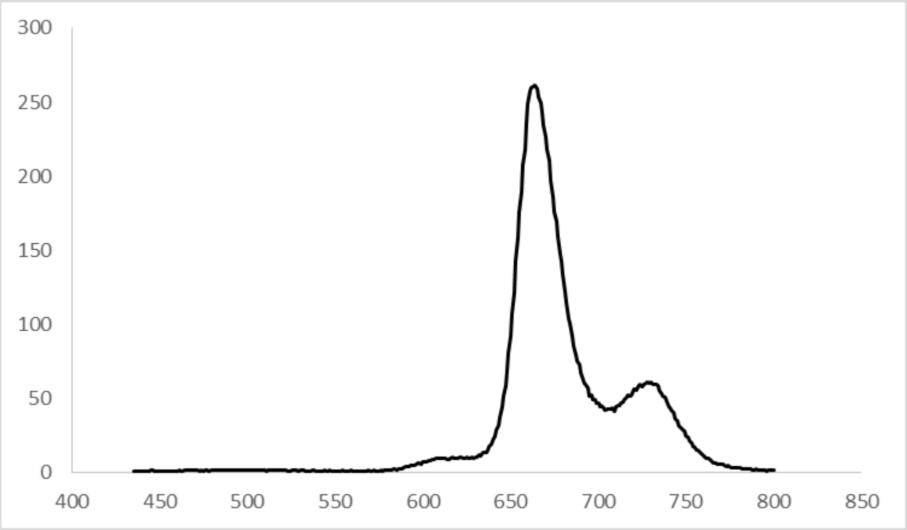

Figure S41. Emission spectrum of **4f** at room temperature (excitation at λ = 420 nm)

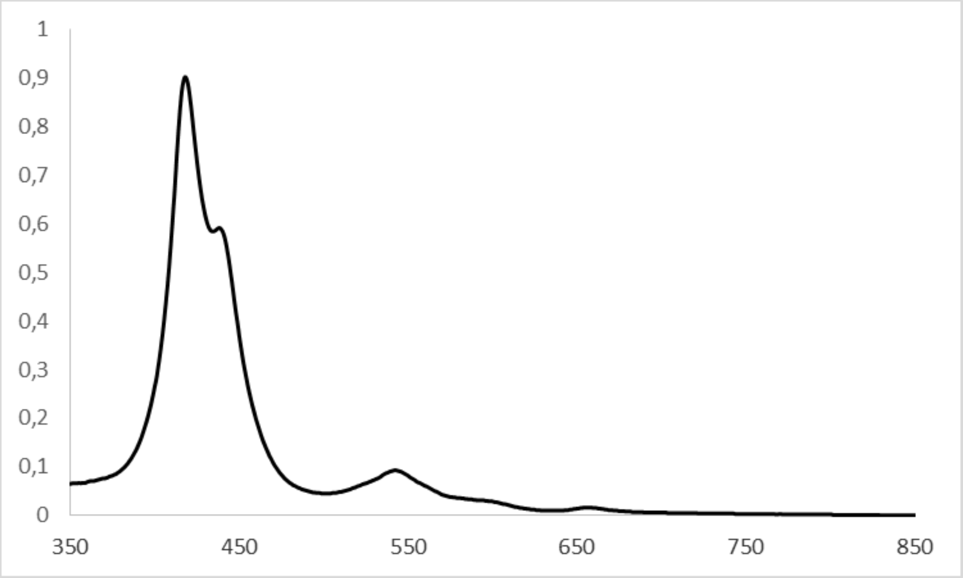

Figure S42. UV–vis absorption spectrum of **4g**.

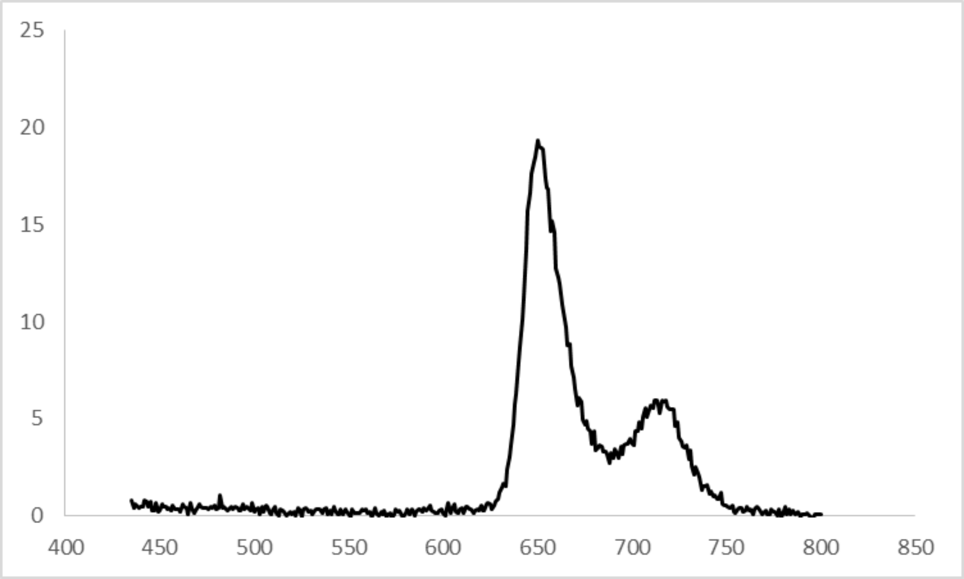

Figure S43. Emission spectrum of **4g** at room temperature (excitation at λ = 420 nm)

References

1. Zhang, W.; Xing, L.; Wang, H.; Liu, X.; Feng, Y.; Gao, C. *Langmuir* **2015**, *31*, 4330-4340.

2. Littler, B. J.; Miller, M. A.; Hung, C.-H.; Wagner, R. W.; O'Shea, D. F.; Boyle, P. D.; Lindsey, J. S. *J. Org. Chem.* **1999**, *64*, 1391-1396.
